# Heterologous Expression of Plantaricin 423 and Mundticin ST4SA in *Saccharomyces cerevisiae*

**DOI:** 10.1007/s12602-023-10082-6

**Published:** 2023-05-12

**Authors:** Michelle Rossouw, Rosemary A. Cripwell, Ross R. Vermeulen, Anton D. van Staden, Willem H. van Zyl, Leon M. T. Dicks, Marinda Viljoen-Bloom

**Affiliations:** 1 Department of Microbiology, Stellenbosch University, Private Bag X1, Matieland, 7602 South Africa; 2 Department of Physiological Sciences, Stellenbosch University, Private Bag X1, Matieland, 7602 South Africa

**Keywords:** *Saccharomyces cerevisiae*, Class IIa bacteriocins, Heterologous expression, Antimicrobial peptides

## Abstract

Antimicrobial peptides or bacteriocins are excellent candidates for alternative antimicrobials, but high manufacturing costs limit their applications. Recombinant gene expression offers the potential to produce these peptides more cost-effectively at a larger scale. *Saccharomyces cerevisiae* is a popular host for recombinant protein production, but with limited success reported on antimicrobial peptides. Individual recombinant *S. cerevisiae* strains were constructed to secrete two class IIa bacteriocins, plantaricin 423 (PlaX) and mundticin ST4SA (MunX). The native and codon-optimised variants of the *plaA* and *munST4SA* genes were cloned into episomal expression vectors containing either the *S. cerevisiae* alpha mating factor (MFα1) or the *Trichoderma reesei* xylanase 2 (XYNSEC) secretion signal sequences. The recombinant peptides retained their activity and stability, with the MFα1 secretion signal superior to the XYNSEC secretion signal for both bacteriocins. An eight-fold increase in activity against *Listeria monocytogenes* was observed for MunX after codon optimisation, but not for PlaX-producing strains. After HPLC-purification, the codon-optimised genes yielded 20.9 mg/L of MunX and 18.4 mg/L of PlaX, which displayed minimum inhibitory concentrations (MICs) of 108.52 nM and 1.18 µM, respectively, against *L. monocytogenes*. The yields represent a marked improvement relative to an *Escherichia coli* expression system previously reported for PlaX and MunX. The results demonstrated that *S. cerevisiae* is a promising host for recombinant bacteriocin production that requires a simple purification process, but the efficacy is sensitive to codon usage and secretion signals.

**Supplementary Information:**

The online version contains supplementary material available at 10.1007/s12602-023-10082-6.

## Introduction

With the increase in antibiotic resistance among bacterial strains, there is an urgent need for alternative antimicrobial agents. Some of the most potent and specific antimicrobials are antimicrobial peptides (AMPs), which are small, cationic peptides (10 to 100 amino acid residues) with an amphipathic structure and a net positive charge [1]. Bacteriocins of gram-positive bacteria are classified into three groups based on their size, structure and modifications [2]. Class IIa bacteriocins, also known as pediocin-like bacteriocins (named after pediocin PA-1, the first bacteriocin characterised in this group), are small (usually less than 10 kDa) and heat-stable, with post-translational modifications that are limited to disulphide bond formation [3, 4]. These peptides contain a conserved N-terminal YGNGV motif (pediocin box) that serves as a recognition sequence for the membrane-bound protein receptor (protein IIC (MptC)) of mannose phosphotransferase (Man-PTS) in target cells [5, 6]. Interaction of the AMP with the IIC docking molecule disrupts the Man-PTS system by preventing the transport of sugars required for growth, causing cell membrane permeabilisation and cell death [4, 7].

Class IIa bacteriocins have been investigated mainly for their application as natural food preservatives, such as antimicrobial packaging and coating fresh produce and meat products [8–10]. Peptides from this subclass mainly target related lactic acid bacteria and different species of *Staphylococcus* and *Listeria*. This includes *Listeria monocytogenes*, the deadliest bacterial source of food poisoning, with 30% of infections in high-risk individuals being fatal [2]. However, there is a growing interest in their application as therapeutic antimicrobial agents as they are highly potent at low concentrations, and their narrow specificity reduces their impact on commensal microbiota [2, 11]. Class IIa bacteriocins are also active against vancomycin-resistant enterococci and *Staphylococcus aureus,* among others [12, 13].

The probiotic lactic acid bacteria, *Lactiplantibacillus plantarum* 423 (previously *Lactobacillus plantarum* 423) and *Enterococcus mundtii* ST4SA, produce the class IIa bacteriocins plantaricin 423 (PlaX) and mundticin ST4SA (MunX), respectively. These AMPs are active against various foodborne pathogens, including *Bacillus cereus*, *Clostridium sporogenes*, *Enterococcus faecalis*, *L. monocytogenes* and *S. aureus* [14, 15]. Van Zyl et al. [16] demonstrated the importance of these bacteriocins as probiotic anti-infective mediators in *L. plantarum* 423 and *E. mundtii* ST4SA to inhibit the growth of *L. monocytogenes* in the gastrointestinal tract of infected mice.

Production of class IIa bacteriocins from their natural producer strains requires the expression of several genes clustered into one or more operons [17]. These operons contain genes that encode a pre-peptide, immunity protein, ATP-binding cassette (ABC) transporter, accessory proteins for the extracellular translocation of the peptide, accessory disulphide modification proteins and regulatory proteins [18]. The complex regulatory machinery can limit or cause inconsistent peptide production from the native host [19, 20]. AMP production by the native host can also be expensive as rich and complex medium is required for improved bacterial growth and peptide production [21]. Moreover, several purification steps are required to purify the peptides from complex growth medium, further inflating the cost of large-scale AMP production.

Recombinant gene expression could allow the more cost-effective and consistent production of AMPs with the potential to scale up the production process. *Escherichia coli* is the most widely used host for producing foreign proteins, but this is hampered by the bacterial host’s sensitivity towards AMPs [22]. Furthermore, *E. coli* does not naturally secrete proteins into the extracellular medium, which can lead to insoluble inclusion bodies and the accumulation of non-functional proteins due to misfolding and a high rate of protein degradation by intracellular proteases [23]. Different strategies have been investigated to overcome this, including signal peptides to facilitate secretion and fusion proteins to mask the activity of AMPs, thus reducing AMP toxicity to the producer strain and preventing proteolytic cleavage [24]. Recombinant plantaricin 423 and mundticin ST4SA were expressed in *E. coli* by fusing the mature peptides to a His-tagged green fluorescent protein (GFP) [25]. The fusion proteins reduced the bacteriocins’ toxicity, but additional purification steps increased AMP production costs and reduced the yields.

The yeast *Saccharomyces cerevisiae* offers a promising alternative to produce class IIa bacteriocins as the cells are not sensitive to AMPs. It can also perform post-translational modification of peptides, such as disulphide bond formation catalysed by two enzymes in the endoplasmic reticulum (ER), namely ER oxidoreductin and protein disulphide isomerase (PDI) [26]. The yeast does not require complex growth media and can efficiently secrete peptides extracellularly, thus increasing the protein titers and reducing the need for additional purification steps [27]. Strains of *S. cerevisiae* are used extensively to produce recombinant proteins and enzymes [28, 29], and a few studies reported the expression of bacteriocins in *S. cerevisiae* [30–33]. Schoeman et al. [30] constructed a bactericidal *S. cerevisiae* Y294 strain that expressed the native pediocin PA-1, while Van Reenen et al. [31] heterologously expressed the native plantaricin 423 in *S. cerevisiae* L5366h*.* However, the expression of both bacteriocins was driven by the inducible yeast alcohol dehydrogenase I promoter (*ADH1*_P_) and terminator (*ADH1*_T_), which yielded low titres.

Different strategies could be employed to improve the heterologous expression of these peptides in *S. cerevisiae*. One approach is to implement codon optimisation, which alters the codon usage pattern using synonymous codons to match the codon usage bias of the native host organism [34]. A secretion signal that targets the AMP to the extracellular matrix would simplify purification, avoid peptide degradation by intracellular proteases and reduce peptide misfolding as chaperone proteins direct the peptides for proper folding and secretion [35]. A strong constitutive promoter and terminator could ensure gene transcription throughout the cultivation process, resulting in higher levels of recombinant peptide [36].

This study aimed to develop an optimised expression system for producing class IIa bacteriocins in *S. cerevisiae*, using the *L. plantarum* 423 and *E. mundtii* ST4SA bacteriocins as benchmarks. Codon-optimised and native variants of *plaA* and *munST4*SA were expressed in *S. cerevisiae* Y294 under the control of the constitutive *ENO1* promoter and terminator. Furthermore, the native *S. cerevisiae* mating factor-alpha secretion signal (MFα1) [37] and the *Trichoderma reesei* xylanase 2 secretion signal (XYNSEC) [38] were evaluated for secretion of the peptides to the extracellular medium.

## Materials and Methods

### Strains and Plasmids

The yeast and bacterial strains used and constructed in this study are listed in Table 1, and the plasmids in Table 2.

**Table 1:** Microbial strains used and constructed in this study

**Strains**	**Genotype**	**Reference/Source**
Bacterial strain		
* E. coli* DH5α	*sup*E44 ∆*lac*U169 (Φ80*lacZ*∆M15) *hsd*R17 *rec*A1 *end*A1 *gyr*A96 *thi*-1 *rel*A1	[39]
* E. coli* BL21 (DE3) PlantEx	fhuA2 [lon] *ompT gal* (λ DE3) [dcm] *∆hsdS* pRSF-GFP-PlaX	[25]
* E. coli* BL21 (DE3) MunEx	fhuA2 [lon] *ompT gal* (λ DE3) [dcm] *∆hsdS* pRSF-GFP-MunX	[25]
* L. monocytogenes* EDG-e	Cam^R^	[25]
*S. cerevisiae* strain		
Y294	MATα *lue*2-3112 *ura*3-52 *his*3 *trp*1-289	ATCC* 201,160
Y294[BBH1]	*URA*3 *ENO1*_P_-*ENO1*_T_	[40]
Y294[BBH4]	*URA*3 *ENO1*_P_-*XYNSEC*-*ENO1*_T_	[40]
Y294[MR]	*URA*3 *ENO1*_P_-*MFα1*-*ENO1*_T_	This study
Y294[MFα1-PlaX_Opt]	*URA*3 *ENO1*_P_-*MFα1*-*plaA_Opt*-*ENO1*_T_	This study
Y294[MFα1-MunX_Opt]	*URA*3 *ENO1*_P_-*MFα1*-*munST4SA_Opt*-*ENO1*_T_	This study
Y294[MFα1-PlaX]	*URA*3 *ENO1*_P_-*MFα1*-*plaA*-*ENO1*_T_	This study
Y294[MFα1-MunX]	*URA*3 *ENO1*_P_-*MFα1*-*munST4SA*-*ENO1*_T_	This study
Y294[XYN-PlaX_Opt]	*URA*3 *ENO1*_P_-*XYNSEC*-*plaA_Opt*-*ENO1*_T_	This study
Y294[XYN-MunX_Opt]	*URA*3 *ENO1*_P_-*XYNSEC*-*munST4SA_Opt*-*ENO1*_T_	This study
Y294[XYN-PlaX]	*URA*3 *ENO1*_P_-*XYNSEC*-*plaA*-*ENO1*_T_	This study
Y294[XYN-MunX]	*URA*3 *ENO1*_P_-*XYNSEC*-*munST4SA*-*ENO1*_T_	This study

*_Opt *codon-optimised coding sequence (GenScript; USA), *XYNSEC* *T. reesei xyn2* secretion signal, *MFα1* *S. cerevisiae* mating factor alpha secretion signal, *PlaX *plantaricin 423, *MunX *mundticin ST4SA

*ATCC American Type Culture Collection

**Table 2:** Plasmids used and constructed in this study

**Plasmids**	**Genotype**	**Reference/source**
pRSF-GFP-PlaX	Kan^R^ T7_P_-His-Tag-GFP-PlaX-T7_T_	[25]
pRSF-GFP-MunX	Kan^R^ T7_P_-His-Tag-GFP-MunX-T7_T_	[25]
yBBH1	*bla URA*3 *ENO1*_P_-*ENO1*_T_	[40]
yBBH4	*bla URA*3 *ENO1*_P_-*XYNSEC*-*ENO1*_T_	[40]
pMR	*bla URA*3 *ENO1*_P_-*MFα1*-*ENO1*_T_	This study
yBBH1-MFα1-PlaX_Opt	*bla URA*3 *ENO1*_P_-*MFα1*-*plaA*_Opt-*ENO1*_T_	This study
yBBH1-MFα1-MunX_Opt	*bla URA*3 *ENO1*_P_-*MFα1*-*munST4SA*_Opt-*ENO1*_T_	This study
pMR-PlaX	*bla URA*3 *ENO1*_P_-*MFα1*-*plaA*-*ENO1*_T_	This study
pMR-MunX	*bla URA*3 *ENO1*_P_-*MFα1*-*munST4SA*-*ENO1*_T_	This study
yBBH4-PlaX_Opt	*bla URA*3 *ENO1*_P_-*XYNSEC*-*plaA*_Opt-*ENO1*_T_	This study
yBBH4-MunX_Opt	*bla URA*3 *ENO1*_P_-*XYNSEC*-*munST4SA*_Opt-*ENO1*_T_	This study
yBBH4-PlaX	*bla URA*3 *ENO1*_P_-*XYNSEC*-*plaA*-*ENO1*_T_	This study
yBBH4-MunX	*bla URA*3 *ENO1*_P_-*XYNSEC*-*munST4SA*-*ENO1*_T_	This study
pUC57-MFα1-PlaX_Opt	*bla MFα1*-*plaA*_Opt	GenScript®
pUC57-MFα1-MunX_Opt	*bla MFα1*-*munST4SA*_Opt	GenScript®

*_Opt *codon-optimised coding sequence (GenScript; USA), *XYNSEC* *T. reesei* xyn2 secretion signal, *MFα1* *S. cerevisiae* mating factor alpha secretion signal, *PlaX *plantaricin 423, *MunX *mundticin ST4SA

### Media and Cultivation Conditions

All media components and reagents were sourced from Merck (Darmstadt, Germany) unless stated otherwise. The *E. coli* DH5α strain (Takara Bio Inc., Japan) was used for plasmid propagation; the transformants were maintained and selected for on Luria Bertani (LB) agar containing ampicillin (100 µg/mL) for selective pressure. Transformants were routinely cultured at 37 °C in Terrific Broth (12 g/L tryptone, 24 g/L yeast extract, 4 m/L glycerol, 0.1 M potassium phosphate buffer; pH 7.0) containing 100 µg/mL ampicillin [39]. The *E. coli* BL21 (DE3) PlantEx and MunEx strains, expressing the native *plaA* and *munST4SA* genes, respectively [25], were maintained on Brain Heart Infusion (BHI) agar supplemented with 50 µg/mL kanamycin.

The *S. cerevisiae* Y294 strain served as the host for recombinant gene expression and was maintained on YPD agar (10 g/L yeast extract, 20 g/L peptone, 20 g/L glucose, 20 g/L agar) and routinely cultured in YPD broth at 30 °C. Recombinant strains were selected for and maintained on SC^−URA^ agar (6.7 g/L yeast nitrogen base without amino acids (BD Diagnostic Systems, Maryland, USA), 20 g/L glucose, 1.5 g/L synthetic drop-out medium supplements without uracil (Sigma-Aldrich, Steinheim, Germany) and 20 g/L agar; pH 6.0). All *S. cerevisiae* strains were aerobically cultivated on a rotary shaker (200 rpm) at 30 °C in 125 mL Erlenmeyer flasks containing 20 mL double-strength SC^−URA^ broth (2 × SC^−URA^) as outlined in [41]. Unless stated otherwise, all growth media were inoculated at a final cell count of 1 × 10^6^ CFU/mL.

*Listeria monocytogenes* EDG-e was used as the indicator strain for all antimicrobial activity assays. The strain was maintained on BHI agar supplemented with 7.5 µg/mL chloramphenicol and incubated at 37 °C.

### Construction of Recombinant Strains

#### Gene Design and Synthesis

The active bacteriocin-coding sequences for the native *plaA* and *munST4SA* genes were obtained from plasmids pRSF-GFP-PlaX and pRSF-GFP-MunX [25]. The DNA sequences of the *plaA* and *munST4SA* genes encoding the mature bacteriocins (excluding the leader peptides) were codon-optimised by GenScript® (Piscataway, New Jersey, USA) using the OptimumGene™ algorithm for expression in *S. cerevisiae*. The resulting plasmids, pUC57-MFα1-PlaX_Opt and pUC57-MFα1-MunX_Opt, contained the native *S. cerevisiae* MFα1 secretion signal and the Kex2 and two Ste13 sites for protease cleavage at the N-termini of the peptides.

#### DNA Manipulations

DNA manipulations and *E. coli* transformations were performed using standard protocols [39]. The various native and codon-optimised nucleotide sequences of the peptides were amplified from plasmid DNA with PCR using primers designed for yeast-mediated ligation (YML) (Online Resource 1). The PCR amplifications were performed with the Gene Amp® PCR System 2400 Thermal Cycler (Perkin Elmer, Waltham, Massachusetts, United States) and TaKaRa Ex Taq™ (Takara Bio Inc.) as per the manufacturer’s recommendations. The PCR products were separated with 2% (w/v) agarose gel electrophoresis and isolated using the Zymoclean™ Gel DNA Recovery kit (Zymo Research, Irvine, California, USA).

The expression cassettes were constructed using yBBH1 and yBBH4 as plasmids backbones. Plasmid yBBH1 contains the *ENO1* cassette, whereas yBBH4 has an additional XYNSEC secretion signal of the *Trichoderma reesei* xylanase 2 gene [38] cloned immediately downstream of the *ENO1* promoter (Table 2; Online Resource 2). yBBH4 served as an expression vector for both the native and codon-optimised bacteriocins, whereas only the codon-optimised bacteriocins fused to the MFα1 secretion signal were cloned into yBBH1. *Nru*I-linearised yBBH4 and *Eco*RI, *Xho*I-digested yBBH1 (restriction enzymes sourced from Inqaba Biotec, Pretoria, South Africa) were separated on a 0.8% (w/v) agarose gel and recovered using the Zymoclean™ Gel DNA Recovery kit (Zymo Research).

The PCR products were co-transformed with the linearised plasmids into electrocompetent *S. cerevisiae* Y294 cells [42] to construct in-frame fusions via YML with the enolase 1 (*ENO1*) promoter and terminator (Online Resource 2). Positive transformants were identified with colony-PCR using gene-specific primers (Online Resource 1) and Sanger sequencing (ABI PRISM™ 3100 Genetic Analyser, Central Analytical Facility, Stellenbosch University) of the plasmid DNA isolated from the yeast strains using the High Pure Plasmid Isolation kit (Roche, Mannheim, Germany).

The MFα1 gene was amplified from the MFα1-PlaX_Opt synthetic gene with the pMR-MFα1 primer set (Online Resource 1) and transformed together with linearised yBBH1 (*Eco*RI + *Xho*I) into electrocompetent *S. cerevisiae* Y294 cells to yield yBBH1-MFα1 (hereafter called pMR, Online Resource 2). The active bacteriocin-coding *plaA* and *munST4SA* genes were amplified from the pRSF-GFP-PlaX and pRSF-GFP-MunX plasmids using the MR-PlaX and MR-MunX primer sets (Online Resource 1), respectively, and transformed together with *Xho*I-linearised pMR to yield plasmids pMR-PlaX and pMR-MunX via YML (Online Resource 2). The gene cassettes are summarised in Online Resource 3, and the nucleotide and amino acid sequences of the secretion signals and various bacteriocins are listed in Online Resource 4.

### Screening for Antimicrobial Activity

The recombinant yeast strains were screened for antimicrobial activity against *L. monocytogenes* EDG-e using agar-overlay and agar well-diffusion assays [30, 43, 44]. The *S. cerevisiae* strains transformed with yBBH1, yBBH4 or pMR (no AMP inserts) served as negative controls. Assays were performed in triplicate, and the average and standard deviation of the diameter of the inhibition zones were determined.

#### Agar-Overlay Assay

Preliminary screening for antimicrobial activity of the various recombinant yeast strains was conducted using the agar-overlay method [30], with a few modifications. Briefly, recombinant strains were grown overnight at 30 °C in test tubes containing 10 mL of SC^−URA^ broth, 2 µL of each culture were spotted onto SC^−URA^ plates and incubated for 72 h at 30 °C. The plates were overlaid with BHI 0.7% (w/v) agar seeded with a 1% (v/v) overnight culture of *L. monocytogenes* EDG-e. After incubation at 30 °C for 18 h, the plates were examined for inhibition zones.

#### Agar Well-Diffusion Assay

Erlenmeyer flasks (125 mL) containing 20 mL 2 × SC^−URA^ broth were inoculated with 1 × 10^6^ cells/mL of the respective recombinant *S. cerevisiae* strain and grown aerobically on a rotary shaker at 200 rpm for 72 h at 30 °C. The cell-free supernatant (CFS) was harvested (1500 × g, 5 min) and filtered through 0.2 µm pore-size low-protein binding non-pyrogenic membranes (syringe filters, Pall Life Sciences, New York, USA). The antimicrobial activity of the CFS was determined using the agar well-diffusion assay [43] by spotting 100 µL of each sample in 6-mm wells that were cut into the surface of BHI 1% (w/v) agar seeded with a 1% (v/v) overnight culture of *L. monocytogenes* EDG-e. All plates were incubated at 37 °C for 18 h and examined for inhibition zones. In addition, the proteinaceous nature of the active supernatant was verified by treating the samples with 10 µg/mL trypsin (in 10 mM TRIS buffer, pH 8) for 1 h at 37 °C, and then determining activity against *L. monocytogenes* EDG-e as described above.

### Bacteriocin Activity Assays

Following the screening for antimicrobial activity, the bacteriocin activity of the recombinant yeast strains was evaluated over 72 h. The strains were cultivated as described above, and 1-mL aliquots of the respective strains were sampled every 24 h. Samples were centrifuged (1500 × g, 5 min), and the supernatant was filtered as described above and assessed for antimicrobial activity [45]. Briefly, a serial two-fold dilution of each sample was made in sterile 1 × phosphate-buffered saline (PBS, pH 7.4), and 100 µL of each diluted sample was tested for activity using the agar well-diffusion assay. The antimicrobial activity of the recombinant strain was expressed as arbitrary units per millilitre (AU/mL), corresponding to the reciprocal of the highest dilution that inhibited the indicator strain [46]. To determine the dry cell weight (DCW) after 72 h of growth, the cells in 1 mL of the supernatant were harvested by centrifugation (1500 × g for 5 min), dried overnight at 60 °C and weighed. Bacteriocin activity assays were performed in three biological repeats, and the average and standard deviations were determined.

### Peptide Analysis Using Tricine-SDS-PAGE

The CFS from the recombinant strains were analysed using tricine SDS-PAGE [47] to confirm the production of the recombinant peptides. Before analysis, CFS was lyophilised for 3 days and dissolved in sterile 1X PBS to achieve a 20-fold concentration. Tricine SDS-PAGE analyses were performed in duplicate using the ultra-low range molecular weight marker (Sigma-Aldrich). One gel was subjected to Coomassie blue staining [47], followed by silver staining [48] to improve the visualisation of protein bands. The other gel was fixed for 20 min in a 25% (v/v) isopropanol, 10% (v/v) acetic acid fixing solution and rinsed thrice for 15 min with sterile Milli-Q water. The gel was then cast in a BHI 0.8% (w/v) agar bilayer (supplemented with 7.5 µg/mL chloramphenicol) seeded with an overnight culture of *L. monocytogenes* EDG-e and incubated overnight at 37 °C to assess antimicrobial activity [44].

### Stability Tests

The antimicrobial activity against *L. monocytogenes* EDG-e was used to assess the stability of the recombinant peptides under different pH and temperature conditions, as outlined in Meng et al. [49]. Briefly, the recombinant strains were cultivated in 2 × SC^−URA^ broth at 30 °C for 72 h and the CFSs harvested (1500 × g for 5 min). The supernatant was filtered as described elsewhere and exposed to a range of pH 2.0 to pH 10.0 (adjusted with either 1 N NaOH or 10 N HCl) and temperatures (4 °C, 30 °C, 37 °C, 60 °C, 80 °C and 100 °C), respectively, for 1 h. After treatment, the pH-treated samples were re-adjusted to the initial pH values, and the temperature-treated samples were allowed to return to room temperature. The antimicrobial activity of the treated samples was determined using the agar well-diffusion assay described above, and the diameter of the inhibition zones was measured. The *S. cerevisiae* Y294[MR] strain and untreated samples were included as controls. Stability tests were performed in duplicate, and an average value and standard deviation were determined for the inhibition zones.

### Scanning Electron Microscopy (SEM)

Scanning electron microscopy was performed as per Reichhardt et al. [50], but with a few modifications. *Listeria monocytogenes* EDG-e was cultured to mid-logarithmic phase; the cells were harvested (2400 × g, 10 min), washed three times with 1X PBS and resuspended to an A_600_ value of 0.2. The bacterial cells were treated with equal volumes of 20-fold concentrated CFS of Y294[MFα1-PlaX_Opt], Y294[MFα1-MunX_Opt] and the Y294[MR] negative control, respectively, at 37 °C for 18 h. The cells were harvested as described elsewhere and washed thrice with 1X PBS. Samples were fixed overnight in 2.5% (v/v) glutaraldehyde with 4% (v/v) paraformaldehyde (PFA) in 0.1 M sodium-cacodylate buffer (pH 7.3) at 4 °C, washed twice in 0.1 M sodium-cacodylate and thrice in sterile Milli-Q water. The cell pellets were dehydrated in a graded ethanol series (30%, 50%, 70%, and 90%) for 15 min each and washed twice with 100% ethanol for 15 min at room temperature. The dried samples were treated with 50% (v/v) hexamethyldisilazane (HMDS) in ethanol for 15 min, followed by 100% HMDS for 15 min and allowed to air-dry overnight. Dried samples were mounted on 10 mm aluminium scanning electron microscopy (SEM) stubs with carbon tape and were coated with gold (10 nm) using a Leica EM ACE200 sputter-coater (Leica Microsystems, Germany) to enhance conductivity. The SEM imaging was completed using a Zeiss Merlin Field Emission SEM (Carl Zeiss Microscopy, Germany) operated at a 2–3 kV accelerating voltage, 89–100 pA beam current and using InLens Secondary Electron (SE) and SE2 detection.

### Peptide Purification

The Y294[MFα1-PlaX_Opt] and Y294[MFα1-MunX_Opt] strains were cultivated as described above, and the CFS was harvested and filter-sterilised. The peptides (henceforth referred to as PlaX_Opt and MunX_Opt) were precipitated from the respective supernatants by adding acetone and trichloracetic acid (TCA) in 1:8:1 solvent ratio for 1 h at − 20 °C. Each mixture was centrifuged at 18,000 × g for 15 min at 4 °C, and the supernatant was discarded. Pellets were washed thrice with ice-cold acetone and dried at room temperature. The dried pellets were then dissolved in 10% (v/v) acetonitrile and tested for activity against *L. monocytogenes* EDG-e using the agar well-diffusion assay.

PlaX_Opt and MunX_Opt were partially purified from the respective active supernatant extractions using reverse-phase High-Performance Liquid Chromatography (HPLC) with the Agilent 1260 Infinity HPLC system and the ZORBAX 300SB-C8 column (4.6 × 150 mm, 5 µm particle size) (Agilent, California, United States). Sample separation was achieved using linear gradient elution from 10% solvent B to 60% solvent B over 25 min (solvent B: acetonitrile + 0.1% (v/v) trifluoroacetic acid (TFA) against solvent A: analytically pure water + 0.1% (v/v) TFA). Elution profiles were monitored at 230 and 214 nm and collected in 1 mL fractions. Acetonitrile was removed from the fractions under vacuum using a SpeedyVac vacuum concentrator (Savant), and the samples were tested against *L. monocytogenes* EDG-e using the agar well-diffusion assay [25].

### Yield Estimation

To calculate the yield of PlaX_Opt and MunX_Opt, the recombinant strains were cultured in 100 mL of 2 × SC^−URA^ broth, and the peptides were harvested and purified as described elsewhere. The HPLC-purified fractions that showed anti-listerial activity were combined, lyophilised and analytically weighed using an XP26 Excellence Plus Micro Balance (Mettler Toledo, Columbus, Ohio, USA). The yields were determined as the mean of three biological repeats in triplicate technical repeats. The purity of the HPLC-purified peptides was confirmed with tricine SDS-PAGE analysis and agar overlays. A concentration range (10 µg–312.5 ng) of bovine serum albumin (BSA) was included as a control for densitometry.

### Liquid Chromatography and Tandem Mass Spectrometry Analysis (LC–MS/MS)

The active fraction that displayed the highest activity from HPLC-purified peptides was selected for LC–MS/MS analysis to confirm the accurate peptide mass and putative disulphide bond location. Liquid chromatography and mass spectrometry analysis were performed as outlined in Swart et al. [51], with a few modifications. A Thermo Scientific Ultimate 3000 RSLC equipped with a C18 trap column (PepMap™; 100 Å pore size, 3 µm particle size, 0 75 µm × 20 mm) and a C18 analytical column (Waters nanoEase M/Z Peptide CSH C18 Column; 130 Å pore size, 1.7 µm particle size, 150 µm × 150 mm) were used for the LC analysis. The solvent system employed was loading: 2% acetonitrile/water containing 0.1% formic acid; Solvent A: water containing 0.1% formic acid and Solvent B: acetonitrile containing 0.1% formic acid. The samples were loaded onto the trap column using the loading solvent at a flow rate of 2 µL/min from a temperature-controlled autosampler set at 7 °C. Loading was performed for 5 min before the sample was eluted onto the analytical column. The flow rate was set to 0.3 µL/min, and the gradient was generated as follows: 5% to 85% Solvent B from 5 to 45 min, 85% to 5% from 45 to 75 min using Chromeleon non-linear gradient 5. Chromatography was performed at 50 °C, and the outflow was delivered to the mass spectrometer through a stainless-steel nano-bore emitter.

Mass spectrometry was performed using an Orbitrap Fusion mass spectrometer (Thermo Scientific, Waltham, Massachusetts) equipped with a Nanospray Flex ionisation source. Data were collected in positive mode with spray voltage set to 1.9 kV and ion transfer capillary set to 275 °C. Spectra were internally calibrated using polysiloxane ions at *m/z* = 445.12. The MS1 scans were performed using the orbitrap detector set at 120 000 resolution over the scan range of *m/z* 375–1500 with automatic gain control (AGC) target at 40,000 and a maximum injection time of 50 ms. Data were acquired in profile mode. The MS2 acquisitions were performed using monoisotopic precursor selection for ions with charges + 2 to + 9 with error tolerance set to approximately 10 ppm. Precursor ions were excluded from fragmentation once for a period of 60 s. Precursor ions were selected for fragmentation in higher-energy collisional dissociation (HCD) mode using the quadrupole mass analyser with HCD energy set to 30%. Fragment ions were detected with the Orbitrap mass analyser set to 30,000 resolution. The AGC target was set to 50,000 and the maximum injection time to 100 ms. The data were acquired in centroid mode. The LC–MS/MS data were processed and analysed using the MZmine 2 software [52] and pLink® 2 [53].

### Minimum Inhibitory Concentration (MIC)

The minimum inhibitory concentrations (MICs) were determined by following the standard broth microdilution method [54]. Briefly, microplates (Greiner CELLSTAR® 96-well plate; Sigma-Aldrich) were loaded with 100 µL of two-fold serial dilutions of the HPLC-purified PlaX_Opt and MunX_Opt in sterile Milli-Q water (starting at 1 mg/mL) and 100 µL of BHI broth. Overnight cultures of *L. monocytogenes* were diluted in BHI broth to obtain *A*_625_ of 0.08–0.1 (representing 1 × 10^8^ CFU/mL), of which 10 µL was added to the respective wells. A positive growth control (*L. monocytogenes* in BHI broth without peptide treatment), a negative control (sterile BHI broth) and a sterility control (BHI broth containing peptide) were included for each MIC assay. The micro-plates were incubated at 37 °C for 8 h with agitation, whereafter the susceptibility of the organisms to the peptides was determined by measuring the *A*_625_ before (*t* = 0) and after incubation (*t* = 8) using an xMark™ Microplate Spectrophotometer (Bio-Rad, San Francisco, USA). The percentage inhibition was determined as the amount of bacteriocin that inhibited growth by at least 90%. The MIC values were reported as the means of two biological repeats in triplicate technical repeats.

### Statistical Analysis

All the data were presented as mean ± standard deviation (SD). For statistical analysis, *t*-tests and one-way analysis of variance (ANOVA) tests were performed, followed by Tukey’s multiple comparison test. Differences with a *p* < 0.05 were considered statistically significant.

### Nucleotide Sequence Accession Numbers

The codon-optimised gene sequences of plantaricin 423 and mundticin ST4SA were submitted to GenBank and assigned the accession numbers OQ703933 and OQ703932, respectively.

## Results

Several industries will benefit from the cost-effective production of AMPs, with possible applications ranging from pharmaceuticals to food preservation. This study aimed to develop an improved expression system for producing class IIa bacteriocins in *S. cerevisiae*. The strategies to improve the production of two bacteriocins included codon optimisation, different secretion signals, and a strong constitutive promotor. Gene cassettes containing various combinations of secretion signals and bacteriocin gene variants were cloned into *S. cerevisiae* Y294, and the strains were evaluated for peptide production and activity.

### Gene Variants and Strain Construction

In this study, the *plaA*, *plaA_Opt*, *munST4SA* and *munST4SA_Opt* genes were cloned into *S. cerevisiae* Y294 to express plantaricin 423 and mundticin ST4SA, respectively (Table 1). The codon optimisation was used to replace rare codons in the DNA sequence with codons preferred by *S. cerevisiae*, thereby increasing the codon frequency (Online Resource 5). The varying distribution of preferred codons in organisms (also known as the codon usage bias) directly affects the translation efficiency of recombinant genes. Based on the codon usage bias for a specific host, different indices can be used to predict the expression of individual gene sequences [34]. For example, the codon bias index (CBI) measures the directional codon bias, i.e. the extent to which a gene uses a subset of ‘optimal’ codons. Alternatively, the codon adaptation index (CAI) measures the usage of preferred codons in a reference set without categorising codons as optimal or non-optimal. Codon optimisation alters the codon usage pattern using synonymous codons, but this strategy does not work for all genes as some of the intrinsic aspects of this technique remain unclear.

The CBI (codon bias index) for *plaA* increased from 0.14 to 0.23 after codon optimisation and for *munST4SA* from 0.05 to 0.59 (Online Resource 6). While the CAI (codon adaption index) predicted a higher increase in expression for *plaA*_Opt than *munST4SA*_Opt, the CBI results predicted an 11.8-fold higher expression for *munST4SA*_Opt and only 1.6-fold for *plaA*_Opt. Plantaricin 423 peptides produced by *S. cerevisiae* strains Y294[MFα1-PlaX], Y294[MFα1-PlaX_Opt], Y294[XYN-PlaX] and Y294[XYN-PlaX_Opt] were refered to as MFα1-PlaX, MFα1-PlaX_Opt, XYN-PlaX and XYN-PlaX_Opt, respectively. Mundticin ST4SA peptides produced by *S. cerevisiae* strains Y294[MFα1-MunX], Y294[MFα1-MunX_Opt], Y294[XYN-MunX] and Y294[XYN-MunX_Opt] were referred to as MFα1-MunX, MFα1-MunX_Opt, XYN-MunX and XYN-MunX_Opt, respectively. The MFα1 and XYNSEC (XYN) secretion signals were indicated within the different gene variants. The Y294[BBH1] control contains an *ENO1* cassette with no secretion signal, the Y294[BBH4] control contains the *ENO1* cassette with the XYNSEC secretion, and Y294[MR] contains the *ENO1* cassette with the MFα1 secretion signal.

### Screening for Antimicrobial Activity

**Fig. 1:**
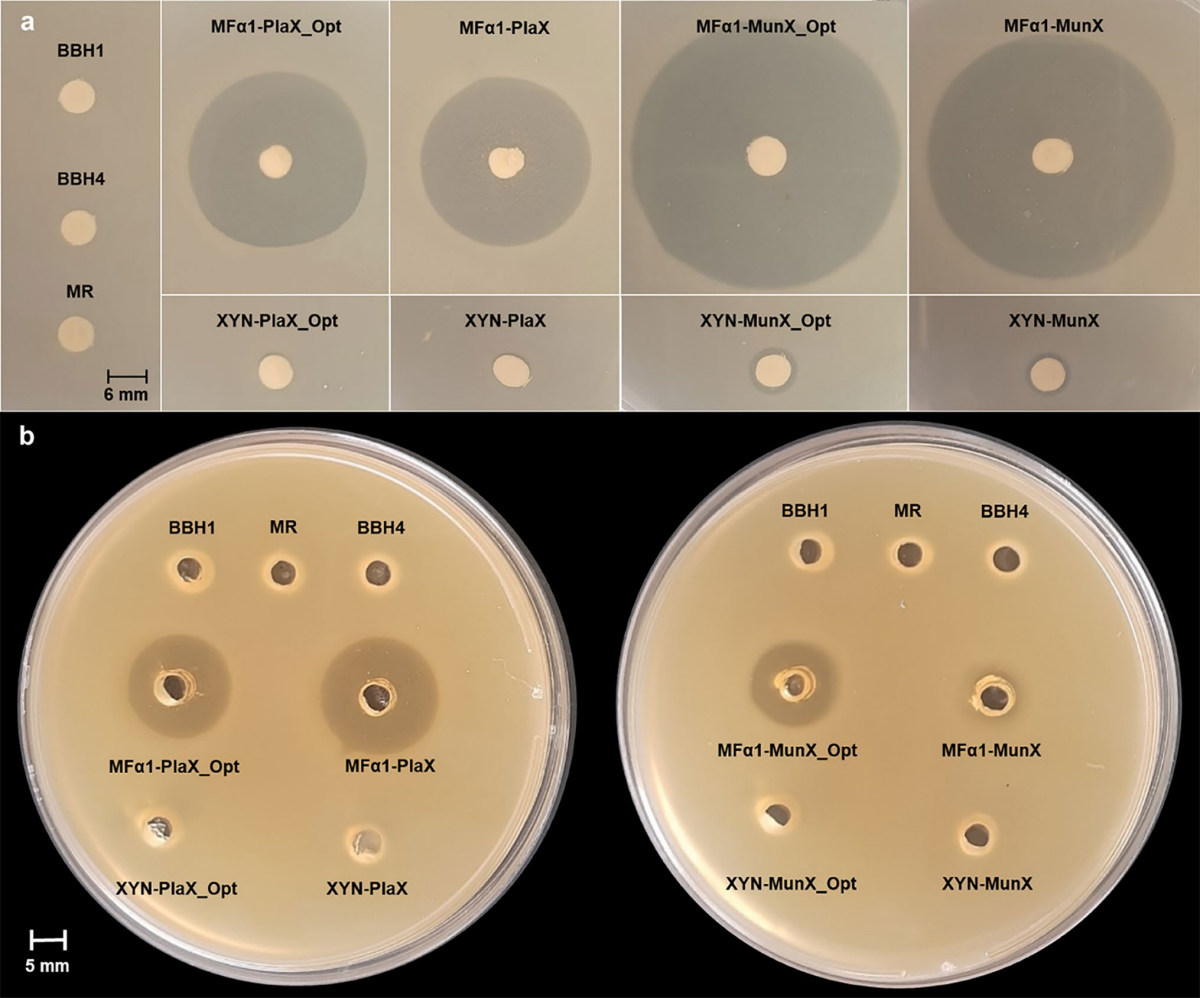
Antimicrobial activity of the recombinant *S. cerevisiae* strains against *L. monocytogenes* EDG-e. **a** Agar-overlay assays with yeast strains and **b** agar well-diffusion assays with CFS from strains producing plantaricin 423 (PlaX) peptide (left) or mundticin ST4SA (MunX) peptide (right). The negative controls are BBH1, BBH4 and MR

The recombinant *S. cerevisiae* strains were evaluated for the production of the plantaricin 423 and mundticin ST4SA peptides using *L. monocytogenes* EDG-e as the indicator organism. In the agar-overlay assay, clear and distinct inhibition zones were observed around the recombinant strains containing either the native and/or codon-optimised peptides with the MFα1 secretion signal (Fig. 1a). Little activity was observed for the strains containing the XYNSEC secretion signal, while no activity was observed for the negative control strains. The agar well-diffusion assays with the CFSs confirmed extracellular anti-listerial activity (clear inhibition zones) for strains containing the MFα1 secretion signal (Fig. 1b). No activity was observed in the supernatant of strains containing the XYNSEC secretion signal, suggesting that the peptides are not secreted into the supernatant.

The results obtained for the agar-overlay and agar well-diffusion assays are summarised in Online Resource 7. The agar-overlay assay showed larger inhibition zones for strains containing the MFα1 secretion signal than those with the XYNSEC secretion signal. The [MFα1-MunX_Opt] and [MFα1-MunX] strains displayed average inhibition zones of 41.3 ± 0.5 mm and 40.3 ± 0.5 mm, respectively, while the [MFα1-PlaX-Opt] and [MFα1-PlaX] strains had average inhibition zones of 27 ± 0.0 mm and 25.7 ± 0.9 mm. In contrast, the [XYN-MunX_Opt], [XYN-MunX], [XYN-PlaX_Opt] and [XYN-PlaX] strains displayed inhibition zones of 6 ± 0.0 mm to 8.3 ± 0.5 mm. The antimicrobial activity for PlaX thus increased by more than 70% and by more than 80% for MunX when expressed with the MFα1 secretion signal relative to the XYNSEC secretion signal. Whereas the CFS from strains containing MFα1 produced inhibition zones of 11.5 ± 2.1 mm for [MFα1-MunX] to 17.5 ± 3.2 mm for [MFα1-PlaX], no activity was observed in the supernatant of strains containing the XYNSEC secretion signal. After treatment with trypsin, the supernatants displayed reduced activity, thus confirming that the activity was linked to protein species and not secreted metabolites (data not shown).

### Quantifying Bacteriocin Activity

**Fig. 2:**
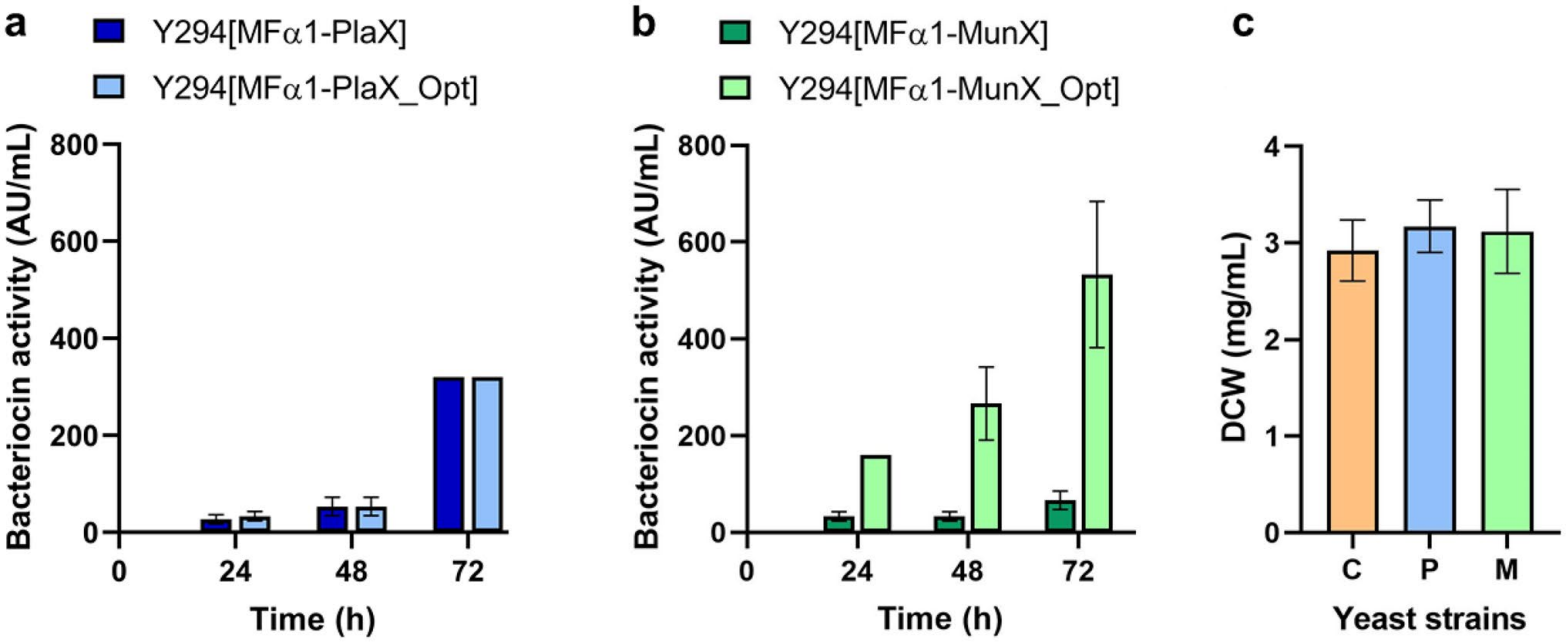
Bacteriocin activity for native and codon-optimised **a** PlaX and **b** MunX strains, and **c** dry cell weight (DCW) for the negative control Y294[MR] (C), Y294[MFa1-PlaX_Opt] (P) and Y294[MFa1-MunX_Opt] (M) strains after 72 h

Bacteriocin activity assays were performed on the supernatant of strains containing the MFα1 secretion signal to quantify their antimicrobial activity (in AU/mL) over 72 h of cultivation (Fig. 2). Antimicrobial activity was observed for all the AMP-producing strains; both the native and codon-optimised PlaX strains reached 320 ± 0.0 AU/mL after 72 h, whereas the native and codon-optimised MunX strains yielded 67 ± 18.86 AU/mL and 533 ± 150.85 AU/mL, respectively, representing a significant eightfold increase (*p* < 0.05). The DCWs of the AMP-producing and negative control strains were comparable, indicating that cell growth was not affected by bacteriocin production.

### SDS-PAGE Analysis

**Fig. 3:**
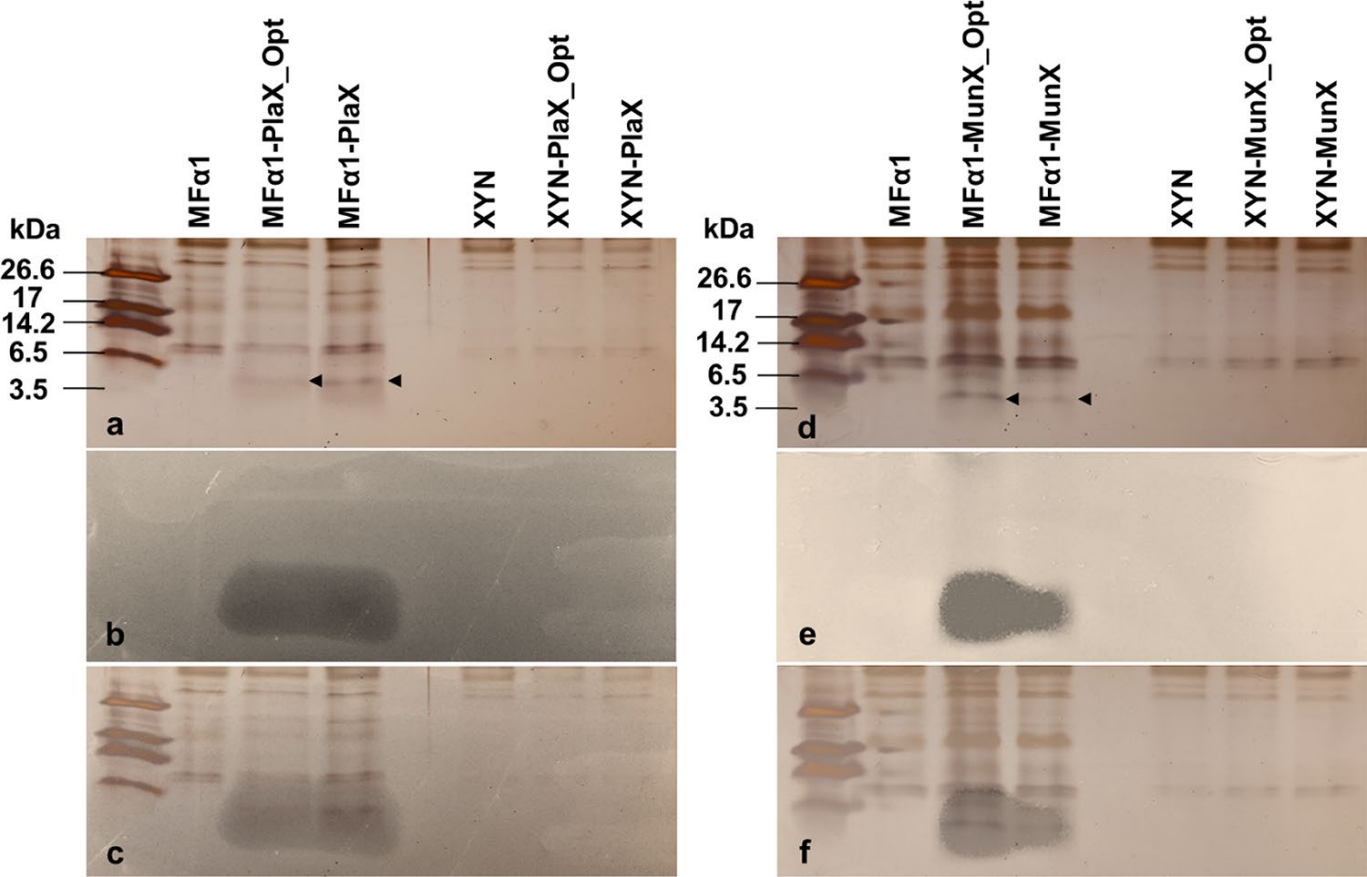
Tricine SDS-PAGE analysis of equal volumes of 20-fold concentrated supernatant containing recombinant **a**–**c** plantaricin 423 (PlaX) or **d**–**f** mundticin ST4SA (MunX) peptides. Images **a** and **d** represent silver-stained gels; **b** and **e** are overlay gels with *L. monocytogenes*; **c** and **f** represent the superimposed gels. Arrows indicate the recombinant PlaX and MunX peptides, respectively. The MFα1 and XYN controls refer to the supernatant of the Y294[MR] and Y294[BBH4] control strains containing the MFα1 and XYNSEC secretion signals, respectively

Tricine SDS-PAGE analysis of 20-fold concentrated supernatants indicated the presence of protein species that correspond to the size of plantaricin 423 (PlaX, 3.9 kDa) and mundticin ST4SA (MunX, 4.2 kDa) in the respective MFα1-containing strains (Fig. 3a, d). No similar size peptides were detected for the negative control or XYNSEC-containing strains. The tricine SDS-PAGE gels were subjected to anti-listerial agar overlay assays, which showed clear zones for the MFα1-containing strains (Fig. 3b, e). The inhibition zones corresponded to the respective peptide size of plantaricin 423 and mundticin ST4SA (Fig. 3c, f).

Based on the results obtained from the antimicrobial activity assays and tricine SDS-PAGE analysis, the yeast strains producing the peptides under the control of the MFα1 secretion signal were selected for further evaluation.

### Stability Assay

The peptides containing the codon-optimised and native nucleotide sequences retained their stability after exposure to temperatures ranging from 4 to 100 °C and a pH of 2.0 to 10.0 (Online Resource 8). For all the strains, antimicrobial activity correlated with an increase in temperature. The ANOVA and Tukey tests revealed significant differences for PlaX_Opt treated at 100 °C relative to treatments at 4 °C, 30 °C and 37 °C, whereas MFα1-PlaX had significantly higher activity at temperatures above 60 °C. The antimicrobial activity of MunX_Opt and MFα1-MunX exposed to temperatures above 80 °C was significantly higher than at the lower temperatures. These results compared well with previous reports on the temperature and pH stability of the native peptides [14].

### SEM Analysis

**Fig. 4:**
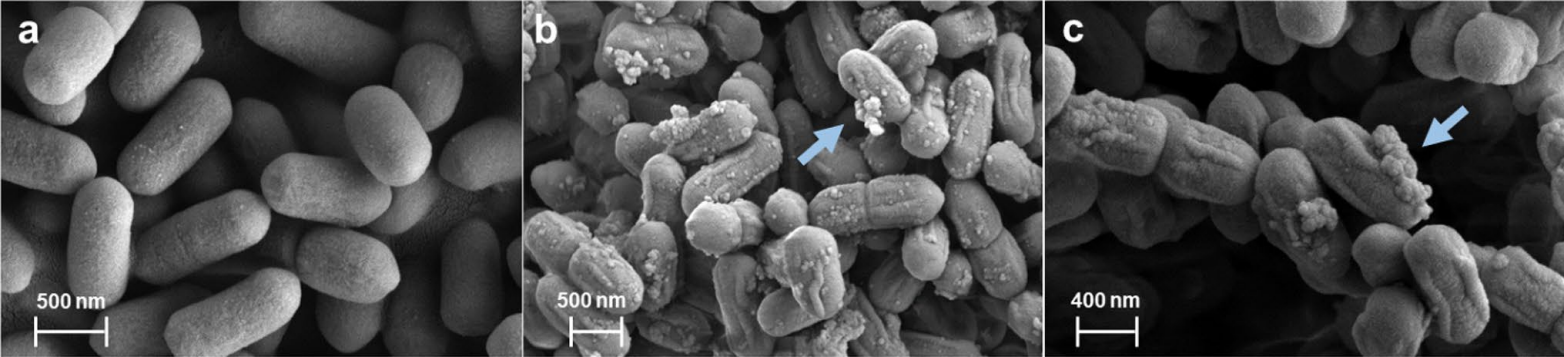
SEM images of *L. monocytogenes* cells after 18 h **a** without treatment, **b** treated with PlaX_Opt and **c** treated with MunX_Opt. The untreated *L. monocytogenes* cells appear smooth and intact, but shrivelled after treatment with PlaX_Opt or MunX_Opt. The arrows indicate intracellular debris released from cells due to pore formation in the cell wall

After the *L. monocytogenes* cells were exposed for 18 h to the concentrated CFS of the Y294[MFα1-PlaX_Opt] and [MFα1-MunX_Opt] strains, changes in the bacterial cell morphology were clearly visible with SEM (Fig. 4). The untreated *L. monocytogenes* cells remained intact and the cell walls appeared smooth (Fig. 4a). After treatment with supernatant from Y294[MFα1-PlaX_Opt] (Fig. 4b) or [MFα1-MunX_Opt] (Fig. 4c), the cells appeared shrivelled, pores formed in the cell wall and intracellular debris were released from the cells, signifying that the *L. monocytogenes* cell membranes were disrupted. After overnight incubation, no growth was observed for the samples treated with either the PlaX_Opt or MunX_Opt peptides, while the untreated sample displayed growth (data not shown).

### Nano-LC–MS and MS/MS Analysis

**Fig. 5:**
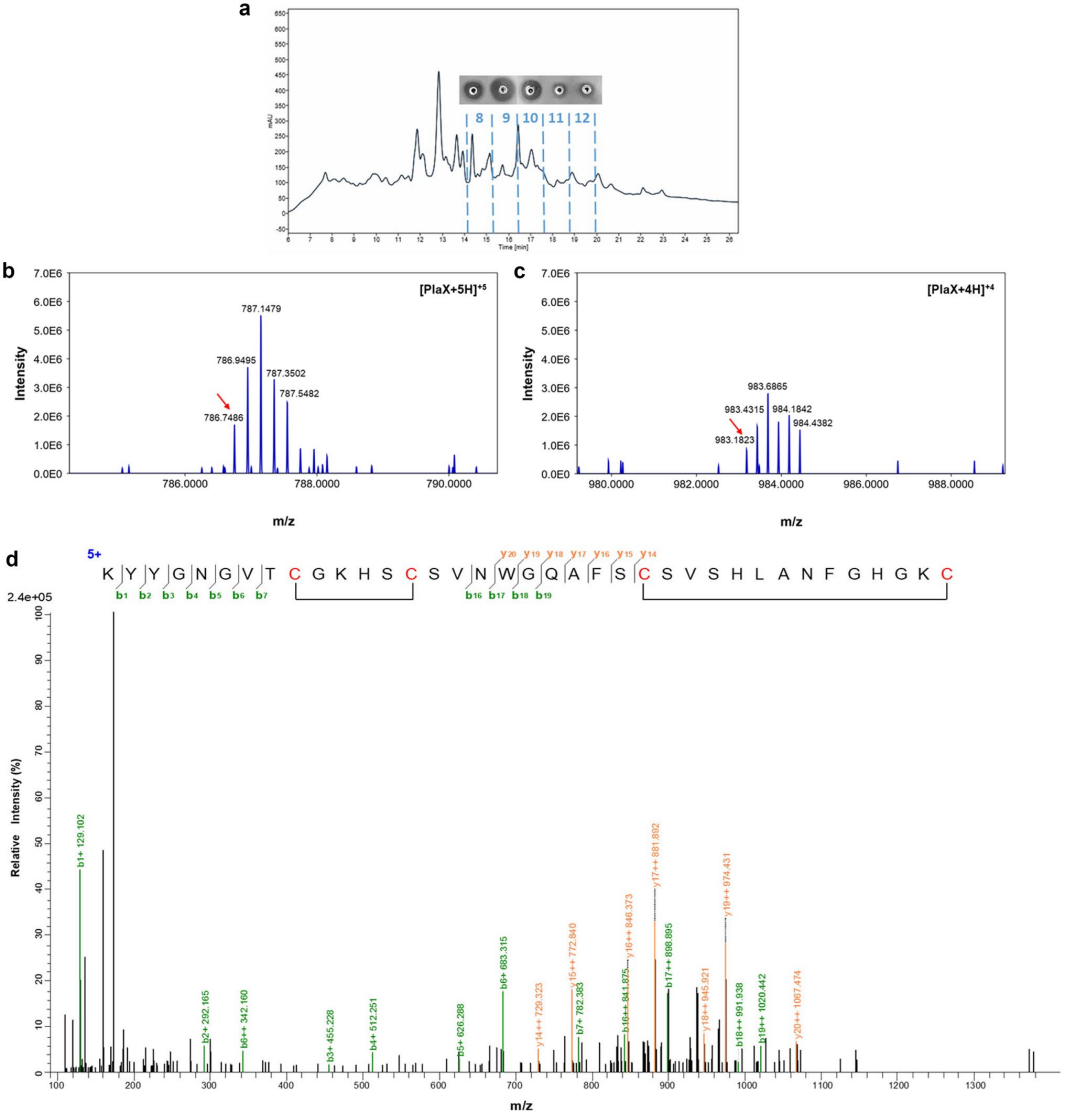
HPLC purification, accurate mass determination and peptide sequence confirmation of PlaX_Opt. **a** HPLC fractionation (C8 column) of PlaX_Opt with anti-listerial activity identified in fractions 8–12, with fraction 9 displaying the highest activity and subsequently analysed with LC–MS/MS. Monoisotopic ions (red arrows) were observed for PlaX_Opt carrying **b** + 5 charges ([M + 5H]^+5^ with an expected *m/z* 786.7493) and **c** + 4 charges ([M + 4H]^+4^ with an expected *m/z* 983.1848). **d** Collision-induced peptide fragmentation spectra confirmed the PlaX_Opt peptide sequence and the two disulphide bonds represented as sections of no fragmentation

**Fig. 6:**
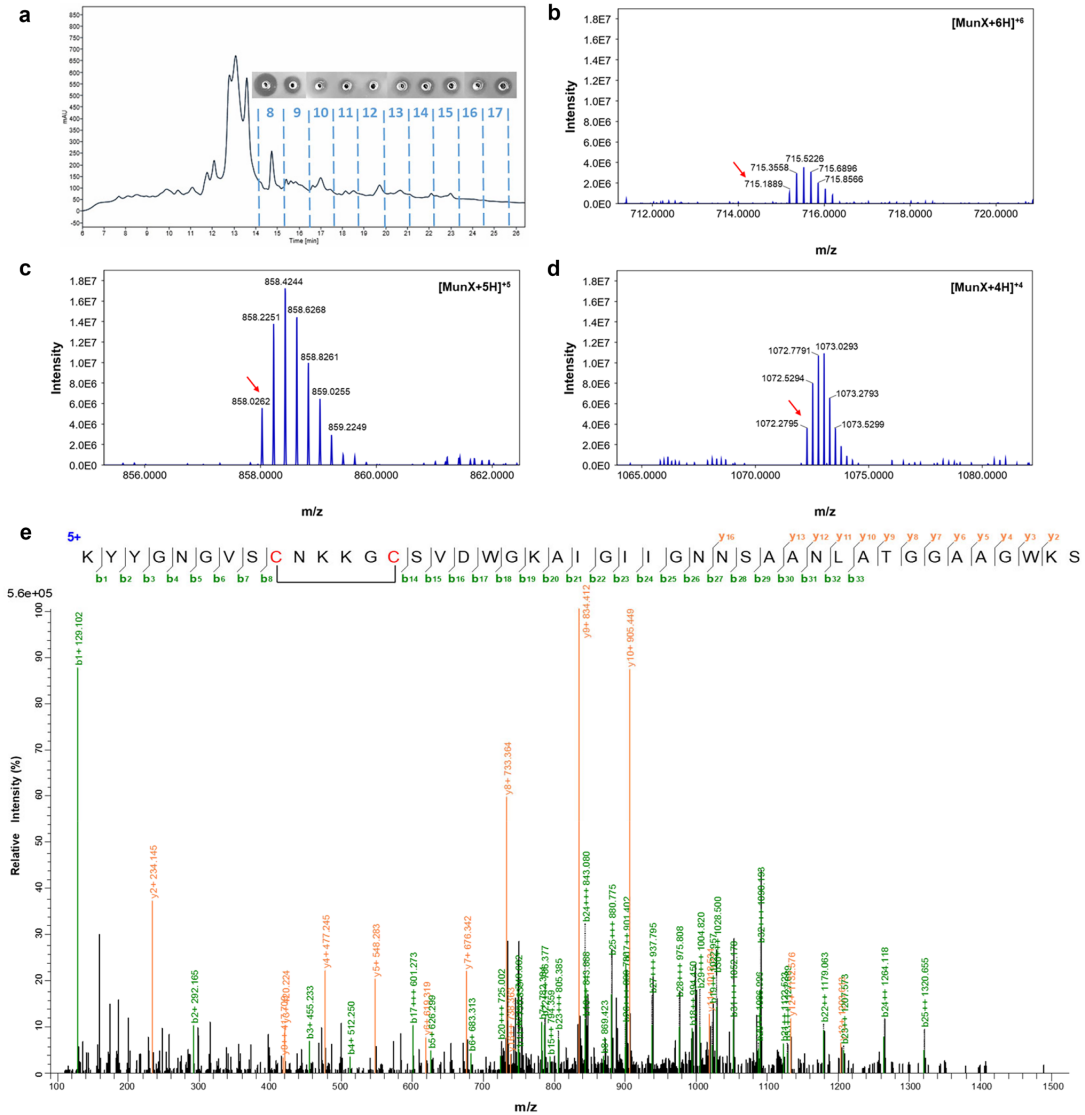
HPLC purification, accurate mass determination and peptide sequence confirmation of MunX_Opt. **a** HPLC fractionation (C8 column) of MunX_Opt with anti-listerial activity identified in fractions 8–17, with fraction 8 displaying the greatest antimicrobial activity and subsequently analysed with LC–MS/MS. Monoisotopic ions (red arrows) were observed for MunX_Opt carrying **b** + 6 charges ([M + 6H]^+6^ with an expected m/z 715.1898), **c** + 5 charges ([M + 5H]^+5^ with an expected m/z 858.0264 and **d** + 4 charges ([M + 4H]^+4^ with an expected m/z 1072.2811). **e** Collision-induced peptide fragmentation spectra confirmed the peptide sequence of MunX_Opt and single disulphide bond represented as the section of no fragmentation

The recombinant peptides were harvested from *S. cerevisiae* Y294[MFα1-PlaX_Opt] and Y294[MFα1-MunX_Opt], respectively, TCA-acetone precipitated and purified using HPLC (with a C8 analytical column). The collected fractions were spot-tested for anti-listerial activity (Figs. 5a and 6a). The HPLC-purified fractions 8–12 of plantaricin 423 and fractions 8–17 of mundticin ST4SA displayed antimicrobial activity. Fractions with the highest anti-listerial activity (fraction 9 of plantaricin 423 and fraction 8 of mundticin ST4SA were further analysed with nano-LC–MS/MS (Figs. 5b–d and 6b–e).

Nano-LC–MS performed on the HPLC-purified active fraction of PlaX_Opt confirmed the presence of a peptide with a mass corresponding to that of the mature plantaricin 423 with two disulphide bonds from the isotopic envelopes of the [M + 5H]^+5^ and [M + 4H]^+4^ species (Fig. 5b, c). The accurate mass measurements closely agree with the theoretical monoisotopic mass of plantaricin 423 (3928.710 Da) (Online Resource 9). A mass corresponding to the mature mundticin ST4SA was confirmed from the isotopic envelopes of the [M + 6H]^+6^, [M + 5H]^+5^ and [M + 4H]^+4^ species (Fig. 6b–d). The accurate mass measurements for MunX_Opt also corresponded to the theoretical monoisotopic mass of mundticin ST4SA (4285.095 Da), equivalent to forming one disulphide bond (Online Resource 9). The monoisotopic parent ion from the peptide envelopes was fragmented and analysed with tandem mass spectrometry (Figs. 5d and 6e). Based on the fractionation patterns, the peptide sequences could accurately be determined with the correct disulphide bond formation, thereby confirming the identities of the recombinantly produced plantaricin 423 and mundticin ST4SA (with a mass error of 10 ppm). The ions observed from the fractionation patterns of plantaricin 423 and mundticin ST4SA are summarised in Online Resource 10 and Online Resource 11, respectively.

### MICs and Yields of HPLC-Purified Peptides

All the active fractions of HPLC-purified PlaX_Opt and MunX-Opt (on the C8 column) were collected and combined, freeze-dried and analytically weighed to determine the peptide yields, with 18.4 ± 3.40 and 20.9 ± 1.59 mg/L obtained for PlaX_Opt and MunX_Opt, respectively (Table 3). Vermeulen et al. [25] reported 12.4 mg/L of purified mundticin and theoretically 12 mg/L of plantaricin produced in *E. coli*. The yields in *S. cerevisiae* were 40.67% and 34.78% higher for active MunX_Opt and PlaX_Opt than *E. coli*.

**Table 3:** Summary of the MIC and yields of the HPLC-purified recombinant peptides

**Peptide**	**Yield (mg/L ± SD)**	**MIC (mg/L ± SD)**	**Molecular mass (g/mol)**	**MIC (nM)**
PlaX_Opt	18.40 ± 3.40	4.65 ± 2.63	3928.70	1183.67 ± 669.59
MunX_Opt	20.90 ± 1.59	0.46 ± 0.0	4285.09	108.52 ± 0.0

*SD* standard deviation

The MICs of the native plantaricin and mundticin against *L. monocytogenes* strain EDG-e were reported to be 12 µM and 10 µM, respectively [55]. These MICs are lower than that observed for the recombinant PlaX_Opt and MunX_Opt against the same strain in this study. However, the native peptides were not 95% pure, and a direct comparison should be treated with caution. The purity of the HPLC-purified peptides in this study was assumed to be > 95% as determined by tricine SDS-PAGE analysis and densitometry (Online Resource 12 and Online Resource 13). Moreover, the respective Y294[MFα1-PlaX_Opt] and Y294[MFα1-MunX_Opt] strains produced 3.96-fold more PlaX_Opt and 44.95-fold more MunX_Opt than required for MIC against *L. monocytogenes*.

## Discussion

In this study, a yeast expression system for the production of bacteriocins in *S. cerevisiae* was developed and optimised using plantaricin 423 and mundticin ST4SA as benchmark peptides. Codon-optimised and native variants of the *plaA* and *munST4SA* genes, encoding the mature plantaricin 423 and mundticin ST4SA peptides, respectively, were cloned in-frame into yeast expression plasmids containing either the MFα1 or XYNSEC secretion signals under the transcriptional control of the *ENO1* promotor and terminator. The plasmids were introduced into *S. cerevisiae* Y294, and the various recombinant strains were evaluated for peptide production and antimicrobial activity.

Results from the agar overlay assays indicated that the inhibition zones observed around the recombinant *S. cerevisiae* strains containing the MFα1 secretion signal were more than 80% larger than for strains containing the XYNSEC secretion signal. However, the agar well-diffusion assays did not indicate activity in the supernatant from the XYNSEC strains, suggesting that the peptides were not secreted extracellularly. This was confirmed when no extracellular proteins were detected for the XYNSEC strains by tricine SDS-PAGE. Although the XYNSEC secretion signal has been used successfully for the secretion of large proteins in *S. cerevisiae* (such as amylases) [41], our results showed that the MFα1 secretion signal was superior for the secretion of the much smaller plantaricin 423 and mundticin ST4SA peptides. It is plausible that the MFα1 secretion signal was evolutionarily adapted to facilitate improved secretion of the small *MFα* mating type peptide [37].

Codon optimisation can enhance gene expression as it alters the codon bias of a foreign protein (bacterial antimicrobial peptides in this case) to match the codon bias of the host organism (e.g. *S. cerevisiae*), but positive results are not guaranteed [56]. The bacteriocin activity assays indicated an eightfold increase in activity for the peptide encoded by the codon-optimised mundticin gene compared to the native gene, which could result from enhanced gene expression. However, the plantaricin 423 peptides produced by the native and codon-optimised genes showed no significant difference in activity. From Online Resource 5, it is clear that the overall relative frequency of codons increased after codon optimisation for both genes, but the codon bias index (CBI) increase for *munST4SA_Opt* was higher than for *plaA_Opt* relative to the native genes. The lower increase in the CBI for *plaA* could explain why no significant difference in the activity of the plantaricin peptides produced by the native and codon-optimised genes was observed. In contrast, codon optimisation of *munST4SA* substantially increased expression in *S. cerevisiae*.

The activity and mode of action of the recombinant peptides were confirmed with SEM analysis, which clearly showed shrivelled bacterial cells and leaking cytoplasm when exposed to the AMPs. The stability of the recombinant peptides was retained after exposure to different pH ranges and temperatures (Online Resource 8), but an increase in activity was observed for both recombinant peptides at higher temperatures. This could result from the degradation of endogenous-produced proteases and proteolytic enzymes at high temperatures, which do not affect the bacteriocins.

The mass spectrometry results confirmed the identities of the recombinant peptides with the correct conformation of disulphide bonds. Furthermore, significant yields were obtained for both peptides (18.4 mg/L plantaricin 423 and 20.9 mg/L mundticin ST4SA). One of the major limiting factors of bacteriocin production is low yields of purified peptides. In a study by Jiang et al. [57], yields of 2–2.5 mg/L plantaricin NC8α and 1.5–2 mg/L plantaricin NC8β were obtained from heterologous expression in *E. coli*. Meng et al. [58] expressed plantaricin Pln1 as a fusion protein with thioredoxin in *E. coli* and obtained 100–110 mg/L fused protein and 9–11 mg/L cleaved peptide. Furthermore, the heterologous co-production of enterocin A and pediocin PA-1 in *Lactococcus lactis* resulted in 27 ng/L and 406 ng/L peptide, respectively [59].

The increased yields and simple purification strategy employed in this study support the notion that yeast may be a better candidate for the expression of plantaricin 423 and mundticin ST4SA. There was no need for fusion proteins as the peptides are not toxic towards the yeast, and the bacteriocins were efficiently secreted into the supernatant, simplifying the harvesting of the peptides. Production of the peptides in *E. coli* requires lysis of the bacterial cells to harvest the peptides, which does not guarantee a 100% peptide recovery rate [25]. Furthermore, the yeast expression system does not require chemical induction (such as IPTG) for the expression and production of bacteriocins, nor does it require protease-mediated liberation of the bacteriocin from a fusion partner [25], which can become costly with the large-scale production of the peptides. Even though bacterial systems can be developed for constitutive peptide production and secretion, antimicrobial peptides can become toxic to bacterial cells. The lack of toxicity against yeast cells, coupled with different options for secretion and constitutive expression, renders yeast advantageous for antimicrobial peptide production. After IMAC purification, mature peptide cleavage, HPLC-purification and lyophilisation, only 12.4 mg/L active mundticin ST4SA was recovered from *E. coli* by Vermeulen et al. [25], compared to 20.9 mg/L mundticin ST4SA produced by *S. cerevisiae*. The yields and purification strategy in this study thus offer significant advantages compared to production in *E. coli*.

The *S. cerevisiae* expression system constructed in this study also outperformed the expression systems reported by Schoeman et al. [30] and Van Reenen et al. [31]. This could be due to codon optimisation of the bacteriocin genes for expression in *S. cerevisiae*, and the use of the constitutive *ENO1* promotor and terminator (instead of the inducible *ADH1* promotor and terminator). However, the production of the peptides in fed-batch bioreactors under controlled conditions should be investigated as this could yield improved biomass production and potentially higher peptide yields. For example, fed-batch fermentation with a 7 g/L/h stable sucrose feeding rate significantly enhanced the production of pediocin SM-1 by *Pediococcus pentosaceus* compared to batch culture [60].

*Pichia pastoris* has always been regarded as the frontrunner for producing bacteriocins from yeast, as the yeast can achieve high concentrations of biomass that result in high protein titers. However, yields reported for purified bacteriocins from *P. pastoris* are not much higher than those obtained from *S. cerevisiae* in this study. Li et al. [61] expressed a hybrid bacteriocin (EF-1) derived from plantaricin E and plantaricin F in *P. pastoris* and obtained 32.65 mg/L of HPLC-purified peptide, while Gutiérrez et al. [62] achieved 22.8 mg/L pure enterocin P from *P. pastoris*. The recombinant expression of bacteriocins in *P. pastoris* mainly involved the *AOX* promotor, which requires induction by methanol, which involves additional costs and complicates purification and downstream applications.

When comparing the activity of the recombinant plantaricin 423 and mundticin ST4SA-producing yeast with the native bacteriocin-producing strains, the MIC of the recombinant *S. cerevisiae* strains were tenfold lower for plantaricin 423 and 92-fold lower for mundticin [55]. Rich and complex growth media is required for the native hosts that renders production expensive and peptide purification difficult. As a result, the purity, and consequently the specific activity, of the native peptides was lower than that achieved for the recombinant peptides produced in this study. The advantages of our expression system include the more consistent production of peptides and simplified purification. Bacteriocin expression by the native host is highly regulated, which can result in inconsistent production [19, 20]. It has been hypothesised that quorum sensing is the primary mode of class IIa bacteriocin regulation, as gene expression depends on producer cell density [63]. Other factors that could influence and further complicate bacteriocin production include temperature and divalent metal cation concentrations [19, 20, 64]. In contrast, the yeast expression system produces bacteriocins constitutively in an active form on relatively cheap media. Furthermore, a simplified purification process was used when compared to the production of plantaricin and mundticin from the native hosts [55]. Due to the simplified *S. cerevisiae* expression system developed in this study, bio-mining and characterisation of novel peptides, as well as peptides that are highly regulated by their native hosts, could be more achievable.

Although improved expression of bacteriocins by the *S. cerevisiae* laboratory strain was demonstrated as a proof of concept, expression of the peptides in an industrial *S. cerevisiae* strain should be investigated for commercial application and up-scaled peptide production. Industrial *S. cerevisiae* strains are better suited to reach high cell densities and withstand harsh industrial conditions such as high temperature, pH and ethanol concentrations. Furthermore, an optimised purification process needs to be developed for the large-scale production of the peptides, as this remains a limiting step.

## Conclusions

The plantaricin 423 and mundticin ST4SA bacteriocins were successfully expressed in *S. cerevisiae*, and the recombinant peptides retained their activity and stability. Different secretion signals and the impact of codon optimisation for expressing the bacteriocin genes in *S. cerevisiae* were investigated. Higher anti-listerial activity was observed for the yeast strain containing the codon-optimised *munST4SA* gene, while no significant difference in activity was observed for the strains containing the codon-optimised *plaA* gene compared to the native bacterial genes. The fusion of the bacteriocin genes to the MFα1 secretion signal allowed for the effective secretion of the bacteriocins into the yeast supernatant, while the peptides apparently remained bound to the yeast cell wall with the XYNSEC secretion signal. Following HPLC-purification, the recombinant *S. cerevisiae* strains yielded 20.9 mg/L of mundticin ST4SA and 18.4 mg/L of plantaricin 423. This is a marked improvement over the yields previously reported for plantaricin 423 and mundticin ST4SA in *E. coli* expression systems, and they required a much simpler purification process.

This study highlights the potential of using *S. cerevisiae* as a host for the heterologous production of antimicrobial peptides. The expression system described in this study could simplify the expression and characterisation of novel peptides, including antimicrobial peptides from other prokaryotes and eukaryotes from underrepresented classes. In addition, future studies should investigate the up-scaled production of the peptides by *S. cerevisiae* in bioreactors for improved yields.

### Supplementary Information

Below is the link to the electronic supplementary material.

Supplementary file1 (DOCX 15 KB)

Supplementary file2 (DOCX 245 KB)

Supplementary file3 (DOCX 82 KB)

Supplementary file4 (DOCX 88 KB)

Supplementary file5 (DOCX 759 KB)

Supplementary file6 (DOCX 13 KB)

Supplementary file7 (DOCX 13 KB)

Supplementary file8 (DOCX 279 KB)

Supplementary file9 (DOCX 13 KB)

Supplementary file10 (DOCX 15 KB)

Supplementary file11 (DOCX 16 KB)

Supplementary file12 (DOCX 735 KB)

Supplementary file13 (DOCX 1509 KB)

## Data Availability

The data sets generated in this study are available on request from the corresponding author.
