# Supplementary material for: Heterologous Expression of Plantaricin 423 and Mundticin ST4SA in Saccharomyces cerevisiae
Source: Probiotics Antimicrob Proteins. 2023 May 12;16(3):845–61. doi: 10.1007/s12602-023-10082-6 (PMC11126478; doi:10.1007/s12602-023-10082-6)
Supplement: Supplementary file 1 — Supplementary file1 (DOCX 15 KB) [file 12602_2023_10082_MOESM1_ESM.docx]

**Online Resource 1**

**Table S1**: PCR primers used in this study with the relevant restriction sites underlined (*Eco*RI = GAATTC; *Xho*I = CTCGAG, *Nru*I = TCGCGA)

| **Coding sequence** | **Amplicon/protein size** | **Primer pair name** | **Primer sequence (5' - 3')** |
| --- | --- | --- | --- |
| PlaX | 111 bp  37 aa | BBH4-PlaX | F: GAACCCGTGGCTGTGGAGAAGCGCTCGCGAAAATACTATGGTAATGGGGTT  R: GGACTAGAAGGCTTAATCAAAAGCTCTCGAGTTAGCACTTTCCATGACCGAA |
|  |  | MR-PlaX | F: GTATCTTTGGATAAAAGAGAGGCTGAAGCTAAATACTATGGTAATGGGGTTACTT  R: GGACTAGAAGGCTTAATCAAAAGCTCTCGAGTTAGCACTTTCCATGACCGAAGTTA |
| MunX | 129 bp  43 aa | BBH4-MunX | F: GAACCCGTGGCTGTGGAGAAGCGCTCGCGAAAATACTACGGTAATGGAGTC  R: GGACTAGAAGGCTTAATCAAAAGCTCTCGAGTTAACTTTTCCAACCAGCTGC |
|  |  | MR-MunX | F: GTATCTTTGGATAAAAGAGAGGCTGAAGCTAAATACTACGGTAATGGAGTCTCAT  R: GGACTAGAAGGCTTAATCAAAAGCTCTCGAGTTAACTTTTCCAACCAGCTGCTCCA |
| PlaX_Opt | 111 bp  37 aa | BBH4-PlaX_Opt | F: GAGGTCGAACCCGTGGCTGTGGAGAAGCGCAAATATTACGGTAATGGTGTTACTT  R: GGACTAGAAGGCTTAATCAAAAGCTCTCGAGTTAACATTTACCATGACCAAAATTT |
| MunX_Opt | 129 bp  43 aa | BBH4-MunX_Opt | F: GAACCCGTGGCTGTGGAGAAGCGCTCGCGAAAGTACTACGGTAACGGTGTTTCTT  R: GGACTAGAAGGCTTAATCAAAAGCTCTCGAGTTATGATTTCCAACCTGCAGCACCA |
| MFα1-PlaX_Opt | 378 bp  126 aa | BBH1-MFα1-PlaX_Opt | F: TCAACACACAAACACTAAATCAAAGAATTCATGAGATTTCCTTCAATTTTTACTG  R: GGACTAGAAGGCTTAATCAAAAGCTCTCGAGTTAACATTTACCATGACCAAAATTT |
| MFα1-MunX_Opt | 396 bp  132 aa | BBH1-MFα1-MunX_Opt | F: TCAACACACAAACACTAAATCAAAGAATTCATGAGATTTCCTTCAATTTTTACTG  R: GGACTAGAAGGCTTAATCAAAAGCTCTCGAGTTATGATTTCCAACCTGCAGCACCA |
| MFα1 | 267 bp  89 aa | MR-MFα1 | F: TCAACACACAAACACTAAATCAAAGAATTCATGAGATTTCCTTCAATTTTTACTG  R: GGACTAGAAGGCTTAATCAAAAGCTCTCGAGAGCTTCAGCCTCTCTTTTATCCAAA |
