# Supplementary material for: Heterologous Expression of Plantaricin 423 and Mundticin ST4SA in Saccharomyces cerevisiae
Source: Probiotics Antimicrob Proteins. 2023 May 12;16(3):845–61. doi: 10.1007/s12602-023-10082-6 (PMC11126478; doi:10.1007/s12602-023-10082-6)
Supplement: Supplementary file 2 — Supplementary file2 (DOCX 245 KB) [file 12602_2023_10082_MOESM2_ESM.docx]

**Online Resource 2**


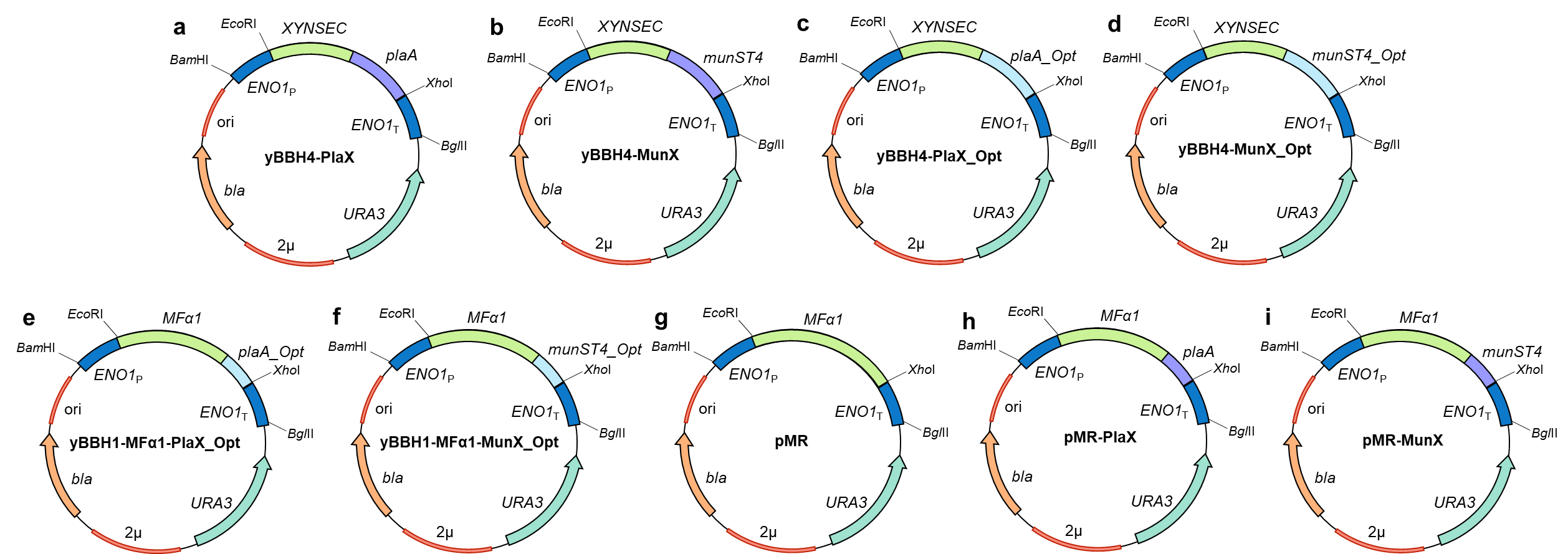


**Fig. S1** Schematic representation of the final plasmid constructs used in this study. The codon-optimised and native *plaA* and *munST4SA* genes were cloned onto plasmid yBBH4 (**a - d**). The *MFα1*-*plaA* (**e**) and *MFα1*-*munST4SA* (**f**) constructs were cloned onto plasmid pBBH1. Plasmid pMR contains the MFα1 secretion signal (**g**) and served as backbone for plasmids pMR-PlaX (**h**) and pMR-MunX (**i**), expressing the native *plaA* and *munST4SA* genes, respectively.
