# Supplementary figures and images for: Heterologous Expression of Plantaricin 423 and Mundticin ST4SA in Saccharomyces cerevisiae
Source: Probiotics Antimicrob Proteins. 2023 May 12;16(3):845–61. doi: 10.1007/s12602-023-10082-6 (PMC11126478; doi:10.1007/s12602-023-10082-6)

**Online Resource 3**


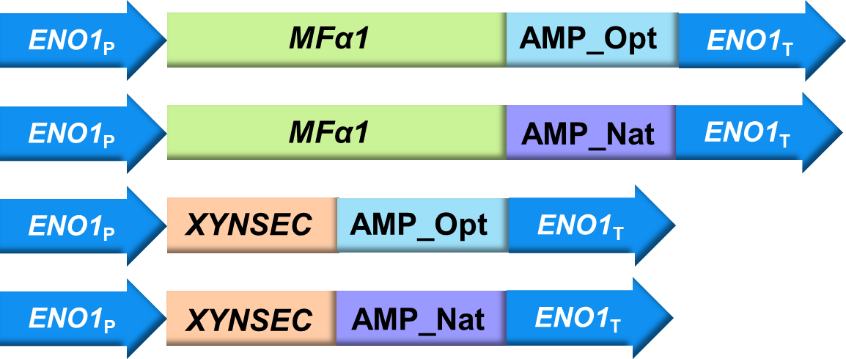


**Fig. S2** Illustration of the different gene constructs designed in this study.

Supplement: Supplementary file 3 — Supplementary file3 (DOCX 82 KB) [file 12602_2023_10082_MOESM3_ESM.docx]
