# Supplementary material for: Heterologous Expression of Plantaricin 423 and Mundticin ST4SA in Saccharomyces cerevisiae
Source: Probiotics Antimicrob Proteins. 2023 May 12;16(3):845–61. doi: 10.1007/s12602-023-10082-6 (PMC11126478; doi:10.1007/s12602-023-10082-6)
Supplement: Supplementary file 4 — Supplementary file4 (DOCX 88 KB) [file 12602_2023_10082_MOESM4_ESM.docx]

**Online Resource 4**


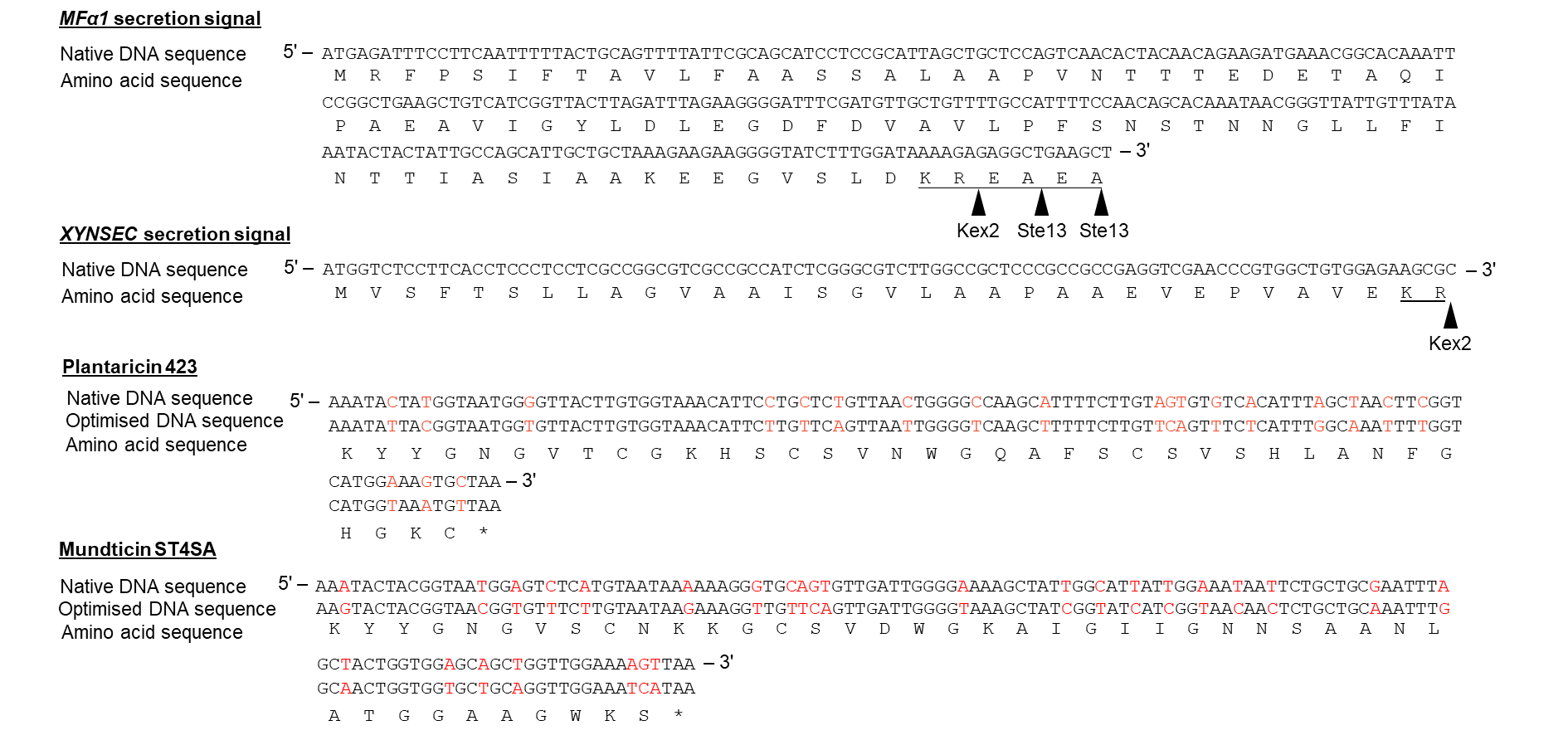


**Fig. S3** Summary of the native nucleotide and peptide sequences of the *MFα1* and *XYNSEC* secretion signals, as well as the native and codon-optimised nucleotide and peptide sequences of plantaricin 423 and mundticin ST4SA. The Kex2 and Ste13 cleavage sites are indicated on the secretion signals. The changes between the native and codon-optimised nucleotide sequences of the bacteriocins are indicated in red.
