# Supplementary material for: Heterologous Expression of Plantaricin 423 and Mundticin ST4SA in Saccharomyces cerevisiae
Source: Probiotics Antimicrob Proteins. 2023 May 12;16(3):845–61. doi: 10.1007/s12602-023-10082-6 (PMC11126478; doi:10.1007/s12602-023-10082-6)
Supplement: Supplementary file 5 — Supplementary file5 (DOCX 759 KB) [file 12602_2023_10082_MOESM5_ESM.docx]

**Online Resource 5**


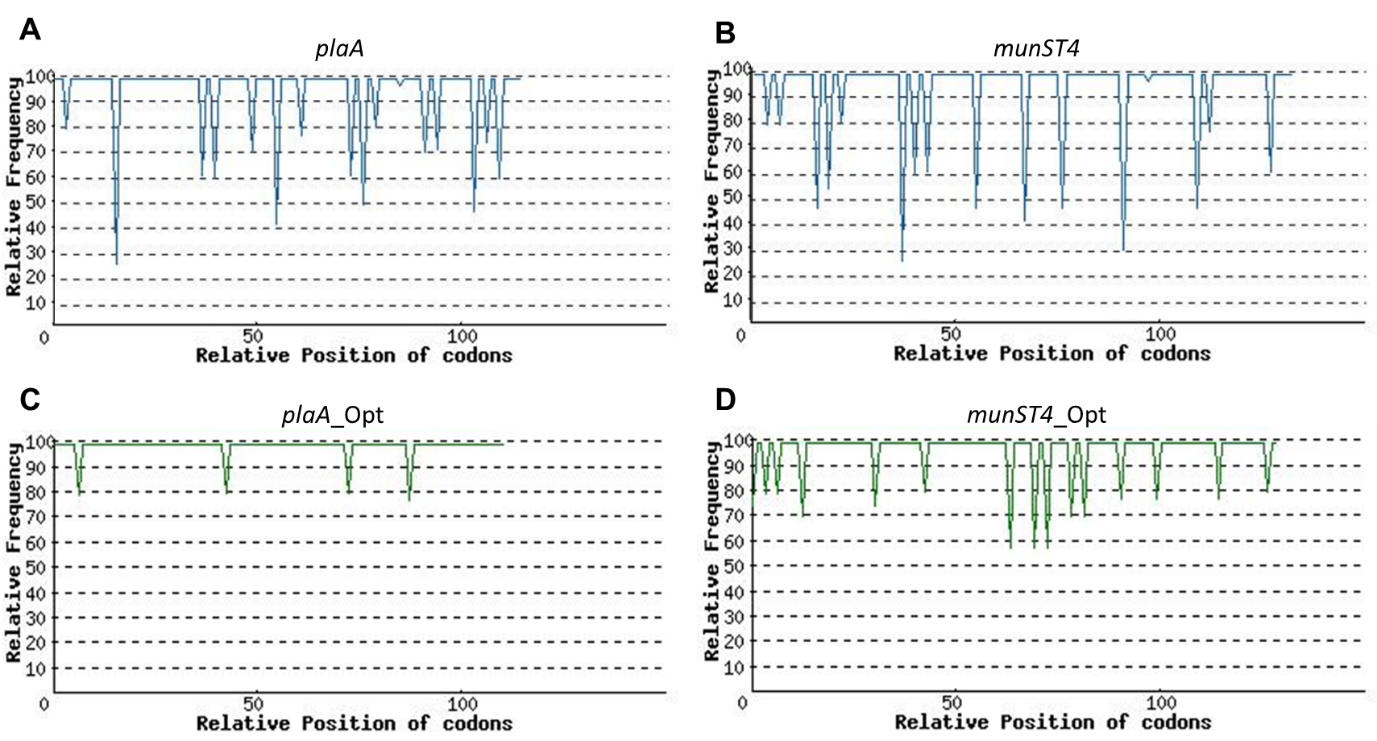


**Fig. S4** The distribution of the relative codon usage frequency along the length of the (A) *plaA*, (B) *munST4SA*, (C) *plaA_Opt* and (D) *munST4SA_Opt* genes expressed in *S. cerevisiae*.
