# Supplementary material for: Heterologous Expression of Plantaricin 423 and Mundticin ST4SA in Saccharomyces cerevisiae
Source: Probiotics Antimicrob Proteins. 2023 May 12;16(3):845–61. doi: 10.1007/s12602-023-10082-6 (PMC11126478; doi:10.1007/s12602-023-10082-6)
Supplement: Supplementary file 6 — Supplementary file6 (DOCX 13 KB) [file 12602_2023_10082_MOESM6_ESM.docx]

**Online Resource 6**

**Table S2** Summary of the bacteriocins used in this study and their codon usage bias

| **Peptide** | **GC content (%)** | | **Codon Bias Index (CBI)** | | **Codon Adaptation Index (CAI)** | | **Host organism** | **Previously expressed in** | **Reference** |
| --- | --- | --- | --- | --- | --- | --- | --- | --- | --- |
|  | Nat | Opt | Nat | Opt | Nat | Opt |  |  |  |
| Plantaricin 423 | 43.36 | 35.42 | 0.14 | 0.23 | 0.81 | 0.97 | *L. plantarum* 423 | *S. cerevisiae*  *E. coli* | Van Reenen et al.. 2003;  Vermeulen et al., 2020 |
| Mundticin ST4SA | 39.02 | 42.57 | 0.05 | 0.59 | 0.80 | 0.89 | *E. mundtii* ST4SA | *E. coli* | Vermeulen et al.. 2020 |
