# Supplementary material for: Heterologous Expression of Plantaricin 423 and Mundticin ST4SA in Saccharomyces cerevisiae
Source: Probiotics Antimicrob Proteins. 2023 May 12;16(3):845–61. doi: 10.1007/s12602-023-10082-6 (PMC11126478; doi:10.1007/s12602-023-10082-6)
Supplement: Supplementary file 7 — Supplementary file7 (DOCX 13 KB) [file 12602_2023_10082_MOESM7_ESM.docx]

**Online Resource 7**

**Table S3** The antimicrobial activity of the recombinant *S. cerevisiae* Y294 strains against *L. monocytogenes* EDG-e

|  | **Diameter of inhibition zone (mm ± SD)** | |
| --- | --- | --- |
| ***S. cerevisiae* strains** | **Agar overlay** | **Agar well diffusion** |
| Y294[MFα1-PlaX_Opt] | 27.0 ± 0.0 | 16.3 ± 3.1 |
| Y294[MFα1-PlaX] | 25.7 ± 0.9 | 17.5 ± 3.2 |
| Y294[XYN -PlaX_Opt] | 7.3 ± 0.5 | 0.0 |
| Y294[XYN-PlaX] | 6.0 ± 0.0 | 0.0 |
| Y294[MFα1-MunX_Opt] | 41.3 ± 0.5 | 15.8 ± 1.3 |
| Y294[MFα1-MunX] | 40.3 ± 0.5 | 11.5 ± 2.1 |
| Y294[XYN-MunX_Opt] | 8.3 ± 0.5 | 0.0 |
| Y294[XYN-MunX] | 7.3 ± 0.5 | 0.0 |
| Y294[BBH1] | 0.0 | 0.0 |
| Y294[BBH4] | 0.0 | 0.0 |
| Y294[MR] | 0.0 | 0.0 |
