# Supplementary material for: Heterologous Expression of Plantaricin 423 and Mundticin ST4SA in Saccharomyces cerevisiae
Source: Probiotics Antimicrob Proteins. 2023 May 12;16(3):845–61. doi: 10.1007/s12602-023-10082-6 (PMC11126478; doi:10.1007/s12602-023-10082-6)
Supplement: Supplementary file 8 — Supplementary file8 (DOCX 279 KB) [file 12602_2023_10082_MOESM8_ESM.docx]

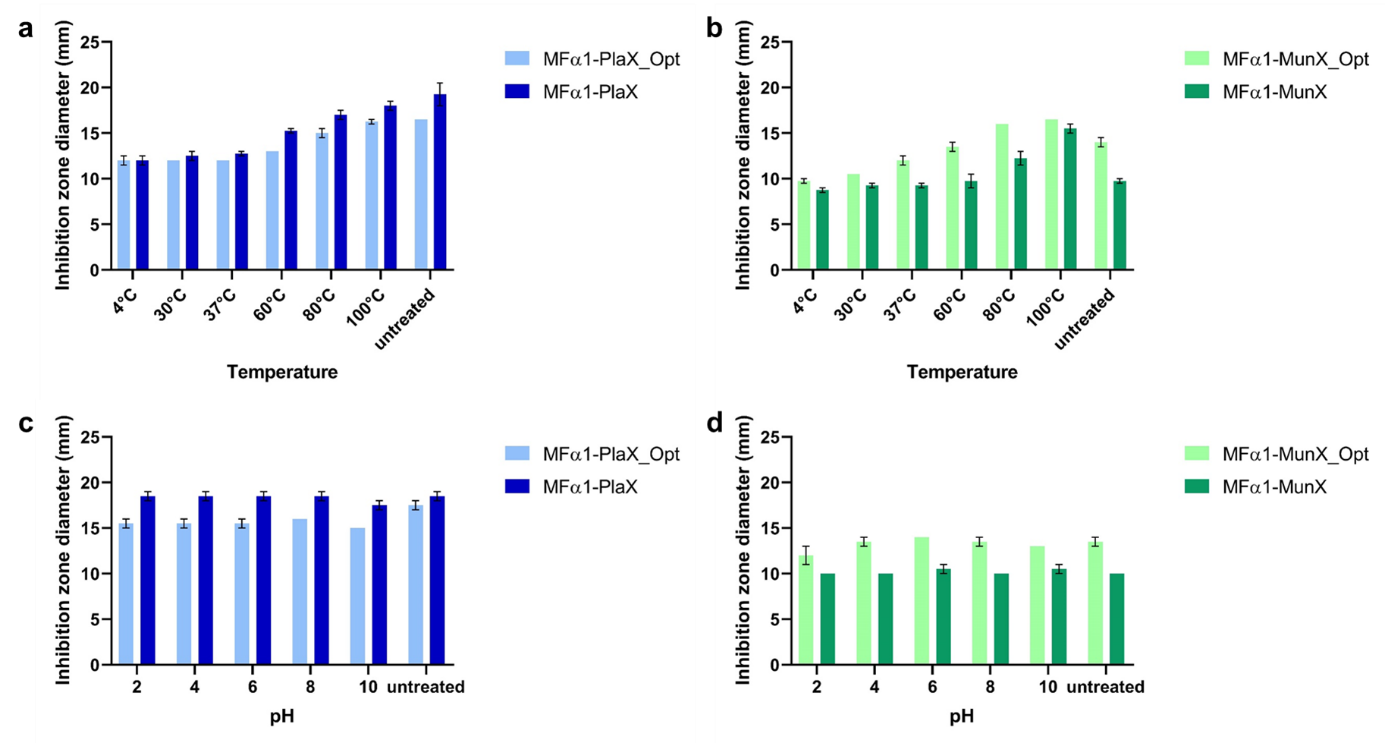
**Online Resource 8**

**Fig. S5** The inhibition zone diameters of PlaX (a and c) and MunX (b and d) after exposure to different temperatures (a and b) and pH values (c and d).
