# Supplementary material for: Heterologous Expression of Plantaricin 423 and Mundticin ST4SA in Saccharomyces cerevisiae
Source: Probiotics Antimicrob Proteins. 2023 May 12;16(3):845–61. doi: 10.1007/s12602-023-10082-6 (PMC11126478; doi:10.1007/s12602-023-10082-6)
Supplement: Supplementary file 9 — Supplementary file9 (DOCX 13 KB) [file 12602_2023_10082_MOESM9_ESM.docx]

**Online Resource 9**

**Table S4**: Summary of the peptides produced by *S. cerevisiae* Y294[MFα1-PlaX_Opt] and Y294[MFα1-MunX_Opt], and their characteristics as per LC-MS

| **Peptide** | **Rt (min)** | **Multiply charged species observed** | **Theoretical**  **m/z** | **Observed**  **m/z** | **mass error (ppm)** | **Theoretical accurate mass (Mr)** | **Observed accurate mass (Mr)** |
| --- | --- | --- | --- | --- | --- | --- | --- |
| Plantaricin 423 | 16.97 | [PlaX+4H]^+4^ | 983.1848 | 983.1823 | 2.54 | 3928.7101 | 3928.7001 |
|  |  | [PlaX+5H]^+5^ | 786.7493 | 786.7486 | 0.89 |  | 3928.7066 |
|  |  |  |  |  |  |  |  |
| Mundticin ST4SA | 16.81 | [MunX+4H]^+4^ | 1072.2811 | 1072.2795 | 1.49 | 4285.0954 | 4285.0889 |
|  |  | [MunX+5H]^+5^ | 858.0264 | 858.0262 | 0.23 |  | 4285.0946 |
|  |  | [MunX+6H]^+6^ | 715.1898 | 715.1889 | 1.26 |  | 4285.0897 |
