# Supplementary material for: Heterologous Expression of Plantaricin 423 and Mundticin ST4SA in Saccharomyces cerevisiae
Source: Probiotics Antimicrob Proteins. 2023 May 12;16(3):845–61. doi: 10.1007/s12602-023-10082-6 (PMC11126478; doi:10.1007/s12602-023-10082-6)
Supplement: Supplementary file 10 — Supplementary file10 (DOCX 15 KB) [file 12602_2023_10082_MOESM10_ESM.docx]

**Online Resource 10**

**Table S5:** Theoretical masses of plantaricin 423 ions. Green and orange text indicate beta and gamma ions, respectively, observed within 10 ppm of the theoretical mass.

| **#** | **b+** | **b++** | **b+++** | **y+** | **y++** | **y+++** |
| --- | --- | --- | --- | --- | --- | --- |
| K(1/*) | 129,1022 | 65,0548 | 43,7056 | * | * | * |
| Y(2/36) | 292,1656 | 146,5864 | 98,06 | 3801,62 | 1901,314 | 1267,878 |
| Y(3/35) | 455,2289 | 228,1181 | 152,4145 | 3638,557 | 1819,782 | 1213,524 |
| G(4/34) | 512,2503 | 256,6288 | 171,4216 | 3475,494 | 1738,25 | 1159,169 |
| N(5/33) | 626,2933 | 313,6503 | 209,4359 | 3418,472 | 1709,74 | 1140,162 |
| G(6/32) | 683,3147 | 342,161 | 228,4431 | 3304,429 | 1652,718 | 1102,148 |
| V(7/31) | 782,3831 | 391,6952 | 261,4659 | 3247,408 | 1624,208 | 1083,141 |
| T(8/30) | 883,4308 | 442,2191 | 295,1485 | 3148,339 | 1574,673 | 1050,118 |
| C(9/29) | Expected disulfide bond | | | | | |
| G(10/28) |  |  |  |  |  |  |
| K(11/27) |  |  |  |  |  |  |
| H(12/26) |  |  |  |  |  |  |
| S(13/25) |  |  |  |  |  |  |
| C(14/24) |  |  |  |  |  |  |
| S(15/23) | 1583,673 | 792,3401 | 528,5625 | 2434,082 | 1217,544 | 812,032 |
| V(16/22) | 1682,741 | 841,8743 | 561,5853 | 2347,05 | 1174,028 | 783,0214 |
| N(17/21) | 1796,784 | 898,8958 | 599,5996 | 2247,981 | 1124,494 | 749,9985 |
| W(18/20) | 1982,864 | 991,9354 | 661,6261 | 2133,938 | 1067,473 | 711,9842 |
| G(19/19) | 2039,885 | 1020,446 | 680,6332 | 1947,859 | 974,4331 | 649,9578 |
| Q(20/18) | 2167,944 | 1084,476 | 723,3194 | 1890,837 | 945,9223 | 630,9506 |
| A(21/17) | 2238,981 | 1119,994 | 746,9984 | 1762,779 | 881,893 | 588,2645 |
| F(22/16) | 2386,049 | 1193,528 | 796,0212 | 1691,742 | 846,3745 | 564,5854 |
| S(23/15) | 2473,081 | 1237,044 | 825,0319 | 1544,673 | 772,8403 | 515,5626 |
| C(24/14) | Expected disulfide bond | | | | | |
| S(25/13) |  |  |  |  |  |  |
| V(26/12) |  |  |  |  |  |  |
| S(27/11) |  |  |  |  |  |  |
| H(28/10) |  |  |  |  |  |  |
| L(29/9) |  |  |  |  |  |  |
| A(30/8) |  |  |  |  |  |  |
| N(31/7) |  |  |  |  |  |  |
| F(32/6) |  |  |  |  |  |  |
| G(33/5) |  |  |  |  |  |  |
| H(34/4) |  |  |  |  |  |  |
| G(35/3) |  |  |  |  |  |  |
| K(36/2) |  |  |  |  |  |  |
| C(*/1) |  |  |  |  |  |  |
