# Supplementary material for: Heterologous Expression of Plantaricin 423 and Mundticin ST4SA in Saccharomyces cerevisiae
Source: Probiotics Antimicrob Proteins. 2023 May 12;16(3):845–61. doi: 10.1007/s12602-023-10082-6 (PMC11126478; doi:10.1007/s12602-023-10082-6)
Supplement: Supplementary file 11 — Supplementary file11 (DOCX 16 KB) [file 12602_2023_10082_MOESM11_ESM.docx]

**Online Resource 11**

**Table S6:** Theoretical masses of mundticin ST4SA ions. Green and orange text indicate beta and gamme ions, respectively, observed within 10 ppm of the theoretical mass.

| **#** | **b+** | **b++** | **b+++** | **y+** | **y++** | **y+++** |
| --- | --- | --- | --- | --- | --- | --- |
| K(1/*) | 129,1022 | 65,0548 | 43,7056 | * | * | * |
| Y(2/42) | 292,1656 | 146,5864 | 98,06 | 4157,992 | 2079,5 | 1386,669 |
| Y(3/41) | 455,2289 | 228,1181 | 152,4145 | 3994,929 | 1997,968 | 1332,314 |
| G(4/40) | 512,2503 | 256,6288 | 171,4216 | 3831,865 | 1916,436 | 1277,96 |
| N(5/39) | 626,2933 | 313,6503 | 209,4359 | 3774,844 | 1887,926 | 1258,953 |
| G(6/38) | 683,3147 | 342,161 | 228,4431 | 3660,801 | 1830,904 | 1220,939 |
| V(7/37) | 782,3831 | 391,6952 | 261,4659 | 3603,779 | 1802,393 | 1201,931 |
| S(8/36) | 869,4152 | 435,2112 | 290,4766 | 3504,711 | 1752,859 | 1168,909 |
| C(9/35) | Expected disulfide bond | | | | | |
| N(10/34) |  |  |  |  |  |  |
| K(11/33) |  |  |  |  |  |  |
| K(12/32) |  |  |  |  |  |  |
| G(13/31) |  |  |  |  |  |  |
| C(14/30) |  |  |  |  |  |  |
| S(15/29) | 1587,704 | 794,3558 | 529,9063 | 2786,422 | 1393,715 | 929,4788 |
| V(16/28) | 1686,773 | 843,89 | 562,9291 | 2699,39 | 1350,199 | 900,4682 |
| D(17/27) | 1801,8 | 901,4034 | 601,2714 | 2600,322 | 1300,664 | 867,4454 |
| W(18/26) | 1987,879 | 994,4431 | 663,2978 | 2485,295 | 1243,151 | 829,103 |
| G(19/25) | 2044,9 | 1022,954 | 682,305 | 2299,215 | 1150,111 | 767,0766 |
| K(20/24) | 2172,995 | 1087,001 | 725,0033 | 2242,194 | 1121,601 | 748,0695 |
| A(21/23) | 2244,032 | 1122,52 | 748,6823 | 2114,099 | 1057,553 | 705,3711 |
| I(22/22) | 2357,117 | 1179,062 | 786,377 | 2043,062 | 1022,035 | 681,6921 |
| G(23/21) | 2414,138 | 1207,573 | 805,3842 | 1929,978 | 965,4925 | 643,9974 |
| I(24/20) | 2527,222 | 1264,115 | 843,0789 | 1872,956 | 936,9817 | 624,9903 |
| I(25/19) | 2640,306 | 1320,657 | 880,7735 | 1759,872 | 880,4397 | 587,2956 |
| G(26/18) | 2697,328 | 1349,167 | 899,7807 | 1646,788 | 823,8977 | 549,6009 |
| N(27/17) | 2811,371 | 1406,189 | 937,795 | 1589,767 | 795,387 | 530,5937 |
| N(28/16) | 2925,413 | 1463,21 | 975,8093 | 1475,724 | 738,3655 | 492,5794 |
| S(29/15) | 3012,445 | 1506,726 | 1004,82 | 1361,681 | 681,344 | 454,5651 |
| A(30/14) | 3083,483 | 1542,245 | 1028,499 | 1274,649 | 637,828 | 425,5544 |
| A(31/13) | 3154,52 | 1577,764 | 1052,178 | 1203,612 | 602,3095 | 401,8754 |
| N(32/12) | 3268,563 | 1634,785 | 1090,192 | 1132,575 | 566,7909 | 378,1964 |
| L(33/11) | 3381,647 | 1691,327 | 1127,887 | 1018,532 | 509,7694 | 340,182 |
| A(34/10) | 3452,684 | 1726,846 | 1151,566 | 905,4475 | 453,2274 | 302,4874 |
| T(35/9) | 3553,731 | 1777,369 | 1185,249 | 834,4104 | 417,7089 | 278,8083 |
| G(36/8) | 3610,753 | 1805,88 | 1204,256 | 733,3627 | 367,185 | 245,1258 |
| G(37/7) | 3667,774 | 1834,391 | 1223,263 | 676,3413 | 338,6743 | 226,1186 |
| A(38/6) | 3738,812 | 1869,909 | 1246,942 | 619,3198 | 310,1635 | 207,1115 |
| A(39/5) | 3809,849 | 1905,428 | 1270,621 | 548,2827 | 274,645 | 183,4324 |
| G(40/4) | 3866,87 | 1933,939 | 1289,628 | 477,2456 | 239,1264 | 159,7534 |
| W(41/3) | 4052,949 | 2026,978 | 1351,655 | 420,2241 | 210,6157 | 140,7462 |
| K(42/2) | 4181,044 | 2091,026 | 1394,353 | 234,1448 | 117,5761 | 78,7198 |
| S(*/1) | * | * | * | 106,0499 | 53,5286 | 36,0215 |
