# Supplementary material for: Heterologous Expression of Plantaricin 423 and Mundticin ST4SA in Saccharomyces cerevisiae
Source: Probiotics Antimicrob Proteins. 2023 May 12;16(3):845–61. doi: 10.1007/s12602-023-10082-6 (PMC11126478; doi:10.1007/s12602-023-10082-6)
Supplement: Supplementary file 12 — Supplementary file12 (DOCX 735 KB) [file 12602_2023_10082_MOESM12_ESM.docx]

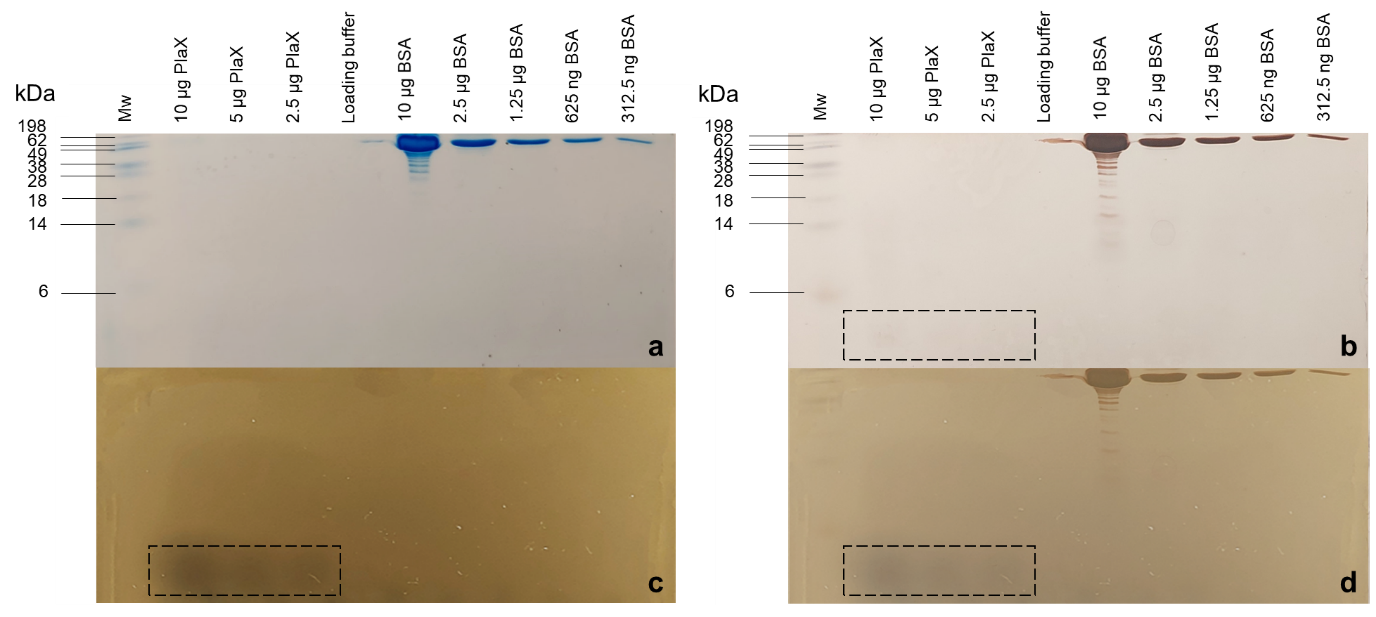
**Online Resource 12**

**Fig. S6:** Tricine SDS-Page analysis and overlay of HPLC-purified plantaricin 423 (PlaX). Image **a** represents the Coomassie blue stained gel and image **b** represents the same gel that was destained and then silver stained. Image **c** represents the antilisterial overlay with inhibition zones indicated with the dashed box. Image **d** represents the superimposed gels. To confirm purity, 10 µg – 2.5 µg of HPLC-purified PlaX was loaded onto the gel. No bands were detected with Coomassie staining; however, a faint band could be detected at 10 µg PlaX. A concentration range (10 µg – 312.5 ng) of bovine serum albumin (BSA) was included as a control for densitometry. No larger bands were detected from the purified peptide samples, indicating at least 95% purity.
