# Supplementary material for: Heterologous Expression of Plantaricin 423 and Mundticin ST4SA in Saccharomyces cerevisiae
Source: Probiotics Antimicrob Proteins. 2023 May 12;16(3):845–61. doi: 10.1007/s12602-023-10082-6 (PMC11126478; doi:10.1007/s12602-023-10082-6)
Supplement: Supplementary file 13 — Supplementary file13 (DOCX 1509 KB) [file 12602_2023_10082_MOESM13_ESM.docx]

**Online Resource 13**


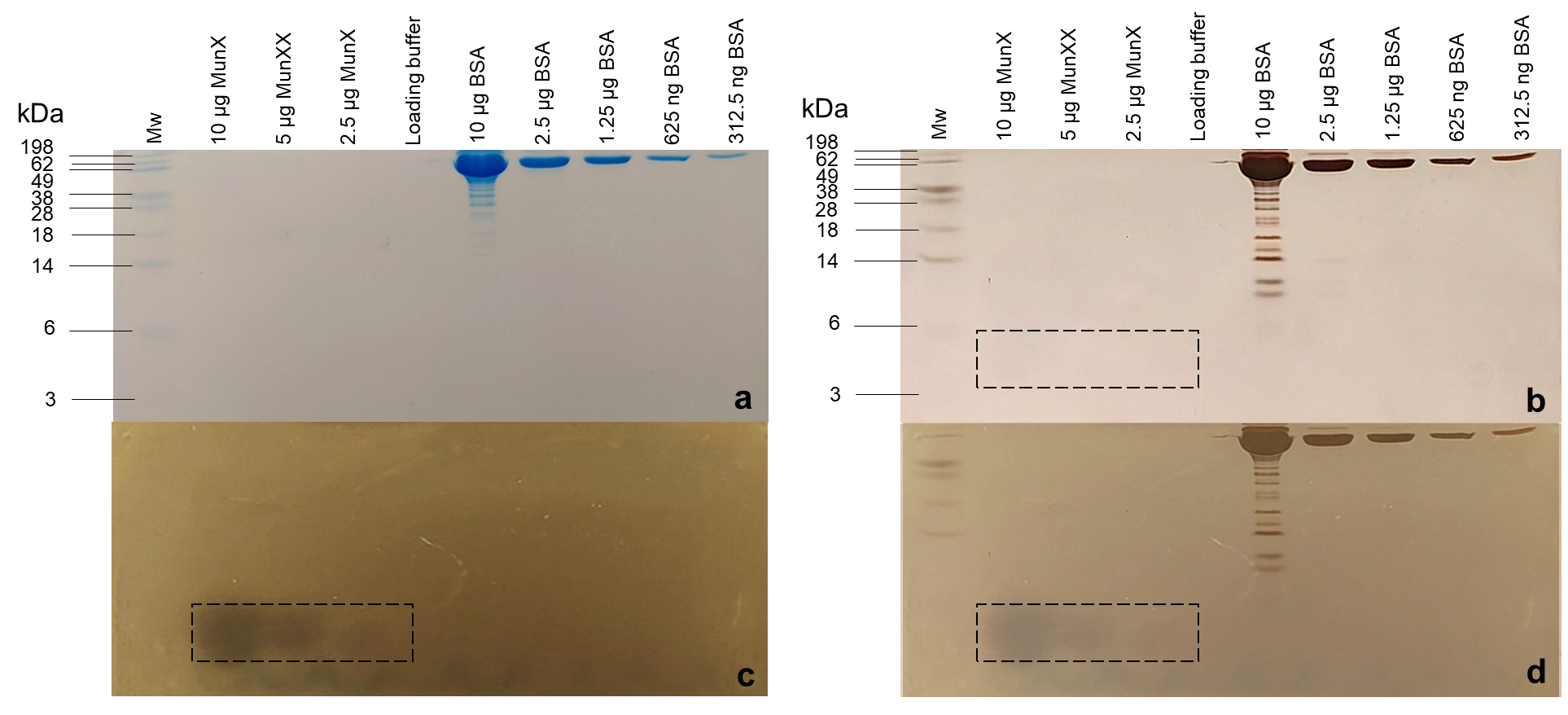
**Fig. S7:** Tricine SDS-Page analysis and overlay of HPLC-purified mundticin ST4SA (MunX). Image **a** represents the Coomassie blue stained gel and image **b** represents the same gel that was destained and then silver stained. Image **c** represents the antilisterial overlay with inhibition zones indicated with the dashed box. Image **d** represents the superimposed gels. To confirm purity, 10 µg – 2.5 µg of HPLC-purified MunX was loaded onto the gel. No bands were detected with Coomassie staining nor silver staining. However, antilisterial activity was detected for MunX. A serial dilution of bovine serum albumin (BSA) of the same concentrations as the peptide was loaded onto the gel as a control. No larger bands were detected from the purified peptide samples, indicating at least 95% purity.
